# Phosphoproteomics of cAMP signaling of *Bordetella* adenylate cyclase toxin in mouse dendritic cells

**DOI:** 10.1038/s41598-017-14501-x

**Published:** 2017-11-24

**Authors:** Jakub Novák, Ivo Fabrik, Irena Linhartová, Marek Link, Ondřej Černý, Jiří Stulík, Peter Šebo

**Affiliations:** 1 Institute of Microbiology of the Czech Academy of Sciences, v.v.i., Prague, Czech Republic; 2 Department of Molecular Pathology and Biology, Faculty of Military Health Sciences, University of Defence, Hradec Kralove, Czech Republic

## Abstract

The adenylate cyclase toxin (CyaA) of the whooping cough agent *Bordetella pertussis* subverts immune functions of host myeloid cells expressing the α_M_β_2_ integrin (CD11b/CD18, CR3 or Mac-1). CyaA delivers into cytosol of cells an extremely catalytically active adenylyl cyclase enzyme, which disrupts the innate and adaptive immune functions of phagocytes through unregulated production of the key signaling molecule cAMP. We have used phosphoproteomics to analyze cAMP signaling of CyaA in murine bone marrow-derived dendritic cells. CyaA action resulted in alterations of phosphorylation state of a number of proteins that regulate actin cytoskeleton homeostasis, including Mena, Talin-1 and VASP. CyaA action repressed mTOR signaling through activation of mTORC1 inhibitors TSC2 and PRAS40 and altered phosphorylation of multiple chromatin remodelers, including the class II histone deacetylase HDAC5. CyaA toxin action further elicited inhibitory phosphorylation of SIK family kinases involved in modulation of immune response and provoked dephosphorylation of the transcriptional coactivator CRTC3, indicating that CyaA-promoted nuclear translocation of CRTC3 may account for CyaA-induced IL-10 production. These findings document the complexity of subversive physiological manipulation of myeloid phagocytes by the CyaA toxin, serving in immune evasion of the pertussis agent.

## Introduction

The Gram-negative coccobacillus *Bordetella pertussis* excels in sophistication of its immunomodulatory action. The bacterium causes the respiratory infectious disease called whooping cough, or pertussis, which can be lethal to unvaccinated infants^1^ and still accounts for an estimated 15 to 50 million cases and ~150,000–300,000 deaths annually world-wide^2^. Among the first cells of the immune system that respond to *B. pertussis* infection are the myeloid phagocytic cells that bear the complement receptor 3 (CR3, the α_M_β_2_ integrin CD11b/CD18 or Mac-1). This includes macrophages, neutrophils and dendritic cells (DCs)^3^. *B. pertussis* employs several mechanisms to subvert their functions. A prominent role in paralysis of these sentinel cells is played by the CR3-binding adenylate cyclase (AC) toxin-hemolysin (CyaA, ACT, or AC-Hly). CyaA is a member of the Repeat In ToXin (RTX) family of leukotoxins^4^ and consists of a cell-invasive adenylyl cyclase (AC) enzyme fused to a pore-forming RTX cytolysin (Hly) moiety^5^. Upon binding to CR3 on cell surface, the toxin translocates its AC domain directly across the plasma membrane into cytosol of phagocytes. There, the AC enzyme is activated by calmodulin and catalyzes unregulated production of a key signaling molecule, the 3′,5′-cyclic adenosine monophosphate (cAMP). Supraphysiological concentrations of cAMP then signal through protein kinase A (PKA) and Exchange Protein directly Activated by cAMP (Epac) pathways^6^ and rapidly annihilate the bactericidal capacities of phagocytes. Signaling of CyaA-produced cAMP provokes massive but unproductive cell ruffling, inhibits opsonophagocytic uptake of bacteria, blocks induction of nitric oxide (NO) production, inhibits NADPH assembly and oxidative burst and induces macrophage apoptosis^6–9^. The molecular details of how CyaA-triggered cAMP signaling interferes with phagocyte functions remain, however, poorly defined. The high specific activity of the CyaA-delivered adenylyl cyclase (AC) enzyme represents, hence, a unique tool for analysis of the impact of cAMP signaling on myeloid cell function in general.

We have used stable isotope labelling by amino acids in cell culture (SILAC)^10^ for quantitative shotgun phosphoproteomic analysis of cAMP signaling resulting from CyaA toxin action on primary mouse bone marrow derived dendritic cells (BMDC). The results reveal that CyaA action causes alteration of phosphorylation of a number of proteins involved in regulation of actin cytoskeleton homeostasis, phagocytosis, translation, chromatin remodeling, IL-10 secretion and tolerogenic DC shaping.

## Materials and Methods

### CyaA toxin preparation

CyaA toxin and its enzymatically inactive CyaA-AC^-^ toxoid were produced in *Escherichia coli* XL-1 Blue cells and purified as previously described^11^, including 60% isopropanol washes of the chromatography resin with bound CyaA, which reduced the endotoxin content of eluted CyaA below 300 IU/mg protein (QCL-1000 Limulus amebocyte lysate assay, Cambrex, East Rutherford, NJ).

### Preparation and SILAC labelling of bone marrow-derived DCs (BMDCs)

The handling of animals was approved by the ethical committees of the Faculty of Military Health Sciences of the University of Defence and of the Institute of Microbiology of the Czech Academy of Sciences. Handling of animals and all experiments were performed in accordance with relevant guidelines and regulations, according to Guidelines for the Care and Use of Laboratory Animals, the Act of the Czech National Assembly, Collection of Laws No. 149/2004, inclusive of the amendments, on the Protection of Animals against Cruelty, and Public Notice of the Ministry of Agriculture of the Czech Republic, Collection of Laws No. 207/2004, on care and use of experimental animals.

The generation and SILAC labelling of C57BL/6 murine BMDCs was carried out as previously described^12^ and outlined in detail in the Supplementary methods section. BMDCs were generated from bone marrow progenitors isolated from femurs and tibias of 6- to 8-week-old female C57BL/6 mice. GM-CSF induces catabolism of ^13^C/^15^N-labelled arginine, yielding heavy arginine-derived proline and ^15^N isotope incorporation, thus skewing estimation of SILAC ratios. Therefore an optimized SILAC-labeling medium was used to suppress these effects, as described earlier^12^.

### Toxin treatment

SILAC-labelled BMDCs were first incubated in D-MEM (1.9 mM Ca^2+^) for 2 hours at 37 °C. ‘Light’ isotope-labelled cells (^12^C6-arginine/^12^C6-lysine) were next treated for 10 or 30 minutes at 37 °C with 100 ng/ml of either CyaA toxin or CyaA-AC^-^ toxoid dissolved in TUC buffer (50 mM Tris-HCl, 8 M urea, 2 mM CaCl_2_, pH 8). Corresponding ‘heavy’ isotope-labelled cells (^13^C6-arginine/^13^C6-lysine) were treated by TUC buffer alone and served as controls for both CyaA and CyaA-AC^-^-treated BMDCs (Supplementary Fig. S1). The whole experiment was performed in biological triplicate and SILAC groups were swapped in one replicate. Cells were next washed in ice-cold PBS and lysed in Lysis buffer (50 mM NH_4_HCO_3_, 1% (w/v) sodium deoxycholate (SDC)) containing a cocktail of phosphatase inhibitors (cocktail set II; Merck). Lysis was accomplished by placing the cell suspension into a boiling water bath for 5 min^13^. After cooling of the lysed suspension to room temperature, the samples were subjected to benzonase treatment (Sigma) for 1 h and cell debris was removed by centrifugation (14,000 *g*, 10 min and 4 °C). Protein concentrations in supernatants were measured using the Micro BCA kit (Thermo Pierce) and the corresponding ‘light’ and ‘heavy’ isotope-labelled lysates were mixed at a 1:1 ratio based on their protein content.

### Phosphoproteomic analysis

Details of sample preparation and data processing are provided in the Supplementary methods. Briefly, proteins in SILAC-labelled BMDC lysates were digested with trypsin and the peptides were fractionated by hydrophilic interaction liquid chromatography (HILIC)^14^. The phosphopeptides were enriched on TiO_2_ resin (GL Sciences), separated by reversed-phase nano-scale LC and analyzed by mass spectrometry on a Q-Exactive MS instrument (Thermo Scientific) operating in data-dependent acquisition (DDA) mode. Data processing, identification and quantitation was performed with MaxQuant software^15,16^ and the MS data were deposited via the PRIDE partner repository^17^ in the ProteomeXchange Consortium database (http://proteomecentral.proteomexchange.org) under the dataset identifier PXD004733. Both WT CyaA and CyaA-AC^–^treated samples were first compared to buffer-treated controls in order to determine the changes in phosphorylation status of each phosphosite. The resulting ratios for individual phosphosites were derived from normalized ratios of the least modified phosphopeptides in a given replicate. Ratio values > 1 then represent dephosphorylation and values < 1 represent increase in phosphorylation. The statistical significance of differences in the phosphorylation status at individual sites was determined by the Global Mean Rank Test^18^. Significantly enriched phosphorylation motifs were assembled using the web-based motif-x algorithm^19^. Assignment to known kinase motifs was performed by Motif Matcher, a web-based online tool from the PHOSIDA posttranslational modification database^20,21^. Alternatively, Fisher’s exact test analysis of kinase motif enrichment was performed for significantly regulated phosphosites by using the Perseus software^22^ with built-in Human Protein Reference Database^23^ as the source of kinase motifs. Functional association between significantly regulated phosphoproteins was visualized using the online-based STRING database (v10; http://string-db.org/; confidence values 0.900 and 0.400, respectively)^24^. Gene Ontology (GO)^25^ enrichment analysis was performed in Cytoscape^26^ using the plugin ClueGO^27^.

## Results

### CyaA triggers PKA and CaMK2-dependent phosphorylation of regulatory proteins

CR3-expressing intraepithelial DCs of myeloid origin (CD11b^+^) are a likely cellular target of the CyaA toxin in the course of *B. pertussis* infection of host airways. We thus assessed the impact of the subversive signaling of CyaA-produced cAMP in DCs by a quantitative phosphoproteomic analysis. Since human monocyte-derived dendritic cells exhibit high inter-donor variation, BMDCs from inbred C57BL/6 mice were used. The cells were labelled *ex vivo* with stable isotopes (SILAC)^12^ and advantage was taken of the use of a non-enzymatic CyaA-AC^-^ toxoid as negative control. CyaA-AC^-^ is unable to elevate cAMP in cells^28,29^ but retains the full capacity to bind the cell surface receptor CR3, to permeabilize cellular membrane and to elicit Ca^2+^ influx into cells and K^+^ efflux from cells, like intact CyaA. This control thus enabled exclusion of phosphorylation changes that were unrelated to cAMP signaling elicited by the CyaA toxin and were due to cell handling and/or cell permeabilization by the pore-forming activity of CyaA. To capture the early cAMP-triggered signaling events and to mimic the physiological concentrations of CyaA encountered by the phagocytes on *B. pertussis*-infected airway mucosa^30,31^, the SILAC-labelled BMDCs (10^6^/ml) were exposed to 100 ng/ml of CyaA or of CyaA-AC^-^ for 10 or 30 minutes.

SILAC-based phosphoproteomic analysis of tryptic digests of 1:1 mixtures of lyzates form ‘heavy’ and ‘light’ isotope-labelled cells was then accomplished as schematically outlined in Supplementary Fig. S1, using biological triplicates and including one label swap condition. This yielded a total of 19,310 identified phosphosites that could be confirmed by MS/MS analysis, including two phosphosites on CyaA itself. From all identified phosphosites, 14,389 were found to be class I sites having a localization probability of >0.75^32^. The observed global phosphorylation pattern of 12,562 serine (87.3%), 1,700 threonine (11.81%) and 127 tyrosine (0.9%) phosphosites (Supplementary Fig. S2B) resembled well the classical radioisotope-based estimates of phosphosite distribution in cells^33^. In total, 6,931 phosphosites were quantified for all three replicates in at least one experimental condition, including the serine residue 393 of CyaA. Compared to buffer control, the treatment of cells with CyaA for 10 or 30 min yielded a significant alteration of the phosphorylation state of the proteome for a total of 313 and 275 sites, respectively. In contrast, upon treatment with CyaA-AC^-^ for 10 min, the alteration of phosphorylation state of only 3 sites passed the significance test. However, phosphorylation status of 56 sites was significantly modulated when BMDCs were exposed to the cell-permeabilizing CyaA-AC^-^ toxoid for 30 min (Supplementary Fig. S2B-C, Supplementary Fig. S3 and Supplementary Table S1).

**Figure 1:**
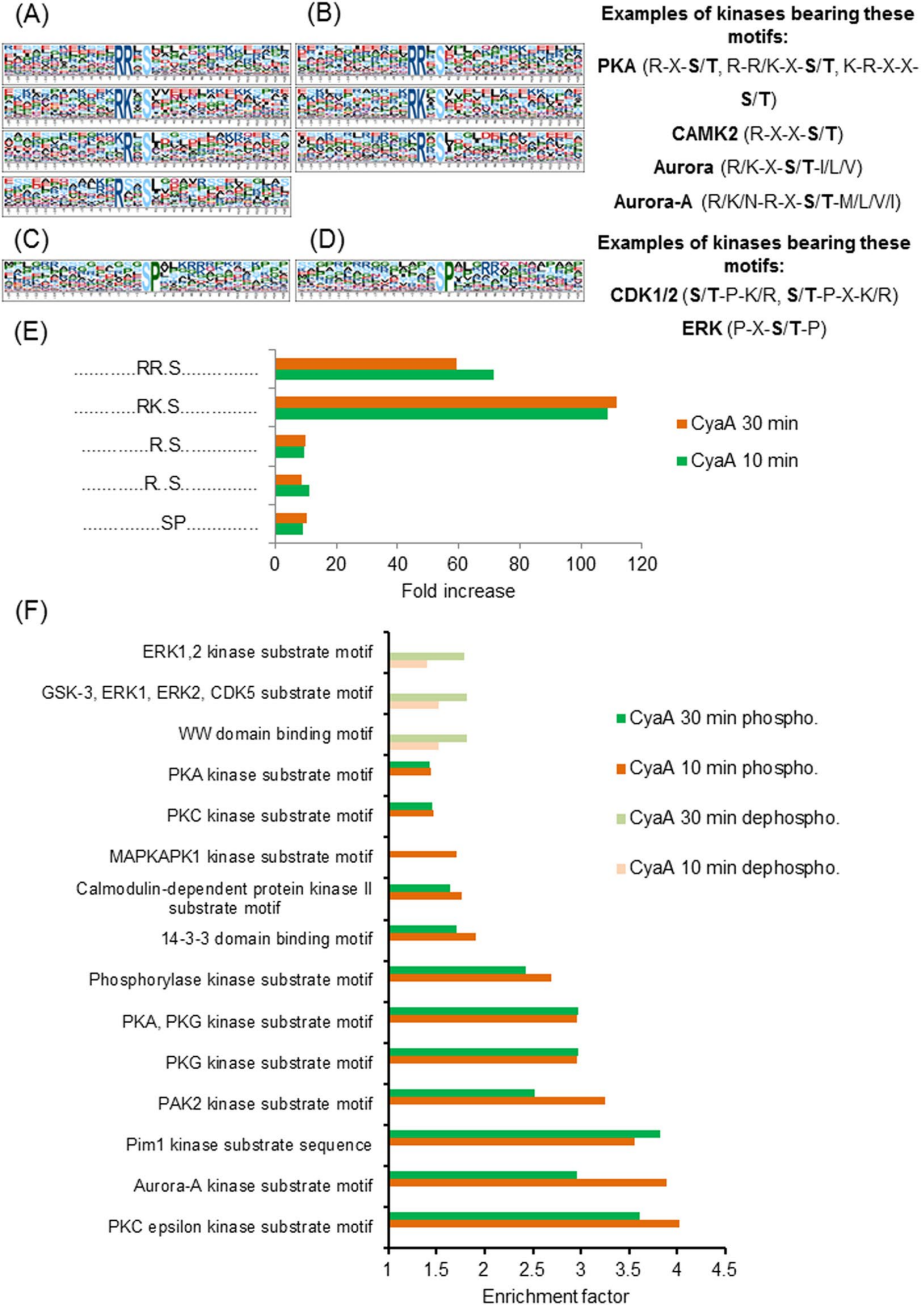
Kinase enrichment analysis of significantly regulated phosphosites. Significantly regulated phosphosites in all replicates for the given experimental condition were identified by Global Mean Rank Test as described in Supplementary Methods. The kinase motifs were next extracted with the web-based motif-x (http://motif-x.med.harvard.edu/) application (Chou & Schwartz, Curr Protoc Bioinformatics. 2011) and assigned to characterized kinases using the PHOSIDA database (http://141.61.102.18/phosida/home.aspx) with the IPI mouse proteome data background (ftp://ftp.ebi.ac.uk/pub/databases/IPI), using a significance threshold of 0.000001 and a minimal occurrence value of the motif set to 20. (**A**) Overrepresented phosphosite motifs extracted as upregulated (phosphorylated) phosphosites by motif-x for samples incubated with CyaA for 10 or (**B**) 30 minutes. Occurrence value of the “..R..S..” motif was 19, hence, just below the set threshold of 20. (**C**) Overrepresented motifs extracted as downregulated (dephosphorylated) phosphosites by motif-x for samples incubated with CyaA for 10 or (**D**) 30 minutes. The “..SP…R..” motif found in the samples treated with CyaA for 30 min exhibited an occurrence number of 19, hence, below the threshold of 20. (**E**) Fold increase of extracted motifs shown in (**A**) and (**B**). Fold increase was calculated as the ratio (foreground matches divided by foreground size) divided by (background matches divided by background size), where the background is the predefined organism background from IPI Mouse Proteome. (**F**) Prediction of kinase-specific phosphorylation sites found among the significantly differentially regulated (both phosphorylated and dephosphorylated) phosphosites in samples treated with CyaA (with localization probability > 0.75 in all replicates in 10 and 30 min samples), compared to buffer-treated controls, by Fisher exact test (Benjamini-Hochberg FDR with the threshold value 0.05). Results are expressed as enrichment factors that are counted using the following formula: (number of hits for particular kinase divided by the number of significantly regulated phosphosites) divided by (number of hits of the particular kinase in the whole given cluster divided by the total size of the dataset). Only kinase enrichment factors > 1 are depicted. Perseus software package for shotgun proteomics data analyzes, version 1.5.1.6, was used for the analysis, employing the Human Protein Reference Database (Release 9).

PKA isoforms are the central cAMP-regulated protein kinases that are likely to mediate most of the immunomodulatory effects of CyaA toxin action^6,7,34,35^. We therefore searched the phosphoproteome data specifically for cAMP-dependent alterations of the phosphorylation state of proteins (Supplementary Table S1) and the overrepresented phosphorylation patterns were extracted using motif-x^19,36^. As expected, the cAMP-activated PKA and Ca^2+^/calmodulin-dependent protein kinase II (CaMK2) were found to be the major kinases mediating cAMP signaling elicited by CyaA (Fig. 1A,B and E). Indeed, the involvement of CaMK2 goes well with the intrinsic capacity of CyaA to bind calmodulin and mediate influx of extracellular Ca^2+^ ions across the plasma membrane of cells^37,38^. Moreover, activation of CaMK2 by the other cAMP effector Epac was previously reported^39^, which suggested a potential indirect role of Epac in phosphoproteome alterations in CyaA-treated cells. The obtained dataset revealed a significant modulation of phosphorylation at sites recognized by the Aurora A kinase^40^ (Fig. 1A,B and E), in line with the previously reported inhibitory impact of CyaA toxin action on mitotic progression and G1/S transition of macrophage cells^41,42^. As further highlighted in Fig. 1C and D, action of CyaA led to the inhibition of cyclin-dependent (CDKs) and extracellular-signal-regulated (ERKs) kinases, yielding an overrepresentation of dephosphorylated proline-directed sites in the dataset.

To corroborate this analysis by an alternative approach, individual phosphosites were annotated for potential kinase motifs according to Human Protein Reference Database^23^ and the enrichment level for regulated sites was next evaluated by Fisher’s exact test using the Perseus software. Following CyaA treatment for 10 or 30 minutes, the PKA, CAMK2 and Aurora A kinase motifs were found to be overrepresented among the increasingly phosphorylated sites, while the Erk1/2 kinase motifs were overrepresented among significantly dephosphorylated sites (Fig. 1C–E). However, BMDC exposure to CyaA for 30 minutes had no effect on the phosphorylation status of the threonine 203 and tyrosine 205 residues in the activation loop of Erk kinases (Supplementary Fig. S4A and B). This indicates that CyaA-produced cAMP signaling activated some phosphatase(s) dephosphorylating the Erk1/2 target motifs.

### CyaA is itself phosphorylated upon translocation into target cells

The 384 N-terminal amino residues of the AC enzyme domain of CyaA are fused to the ‘AC to Hly-linking segment’ (residues 385–485)^43^ that plays an essential role in AC domain translocation across the plasma membrane of cells^44^. The phosphoproteomic analysis revealed a phosphorylation signal of the serine 393 residue of CyaA (Supplementary Table S1, for processed MS/MS spectrum see Supplementary Fig. S5), showing that the N-proximal end of the ‘AC to Hly-linking segment’ penetrates into cell cytosol.

### Adenylyl cyclase enzyme and pore-forming activities of CyaA exert counteracting effects on PI3K-Akt pathway

**Figure 2:**
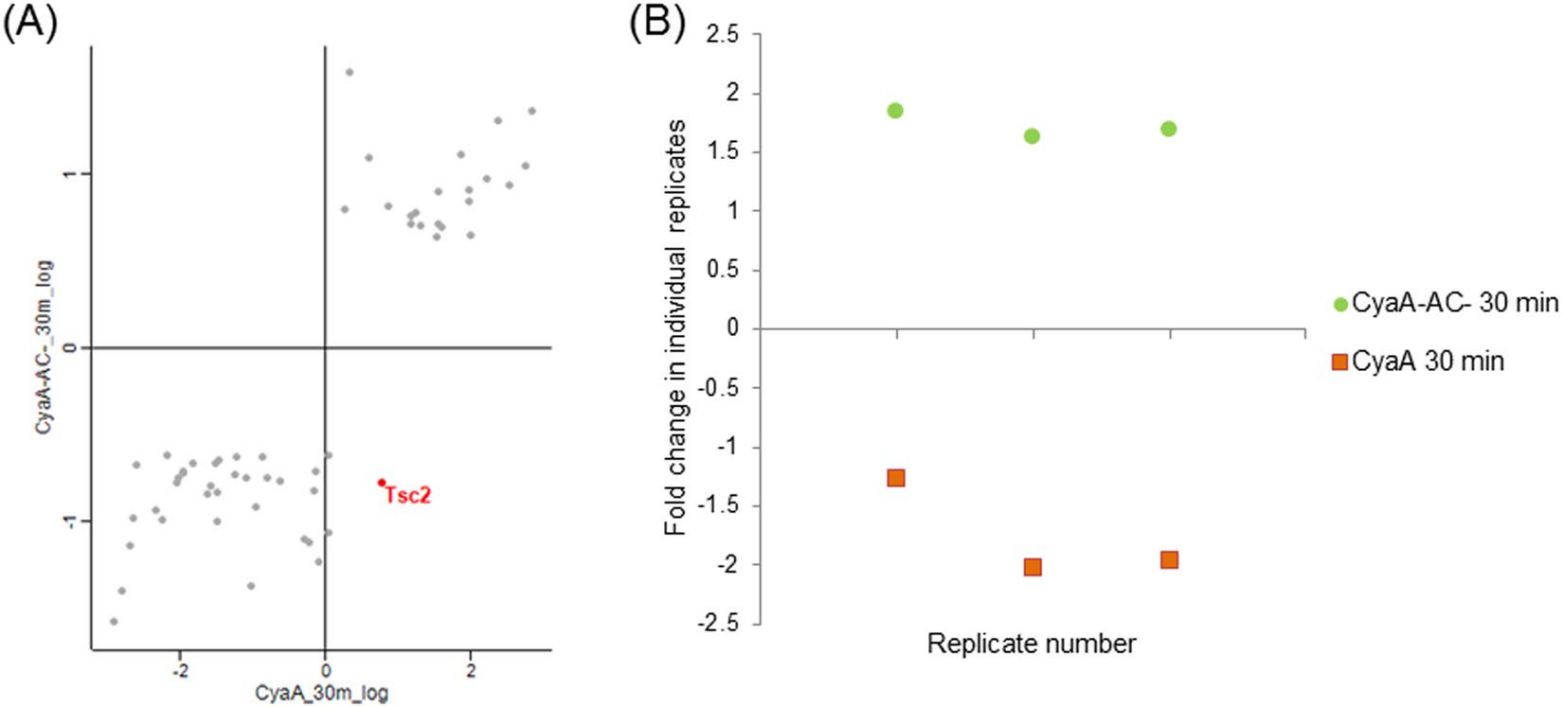
Phosphorylation of tuberin (Tsc2) is oppositely manipulated by the cAMP signaling and pore-forming activities of CyaA. (**A**) Scatter plot of all significantly regulated phosphosites (based on signal ratios of toxin or toxoid-treated samples to buffer-treated samples). Values for the samples treated by CyaA-AC^-^ toxoid for 30 minutes are plotted against the corresponding values for the samples treated by WT CyaA for 30 minutes. Tsc2, showing an inverse correlation between the two conditions, is highlighted. (**B**) Fold change of phosphorylation status (toxin- or toxoid-treated sample *versus* buffer-treated control) of the threonine residue 1465 of Tsc2 for three individual replicates (values > 0 represent phosphorylation, values < 0 represent dephosphorylation).

**Figure 3:**
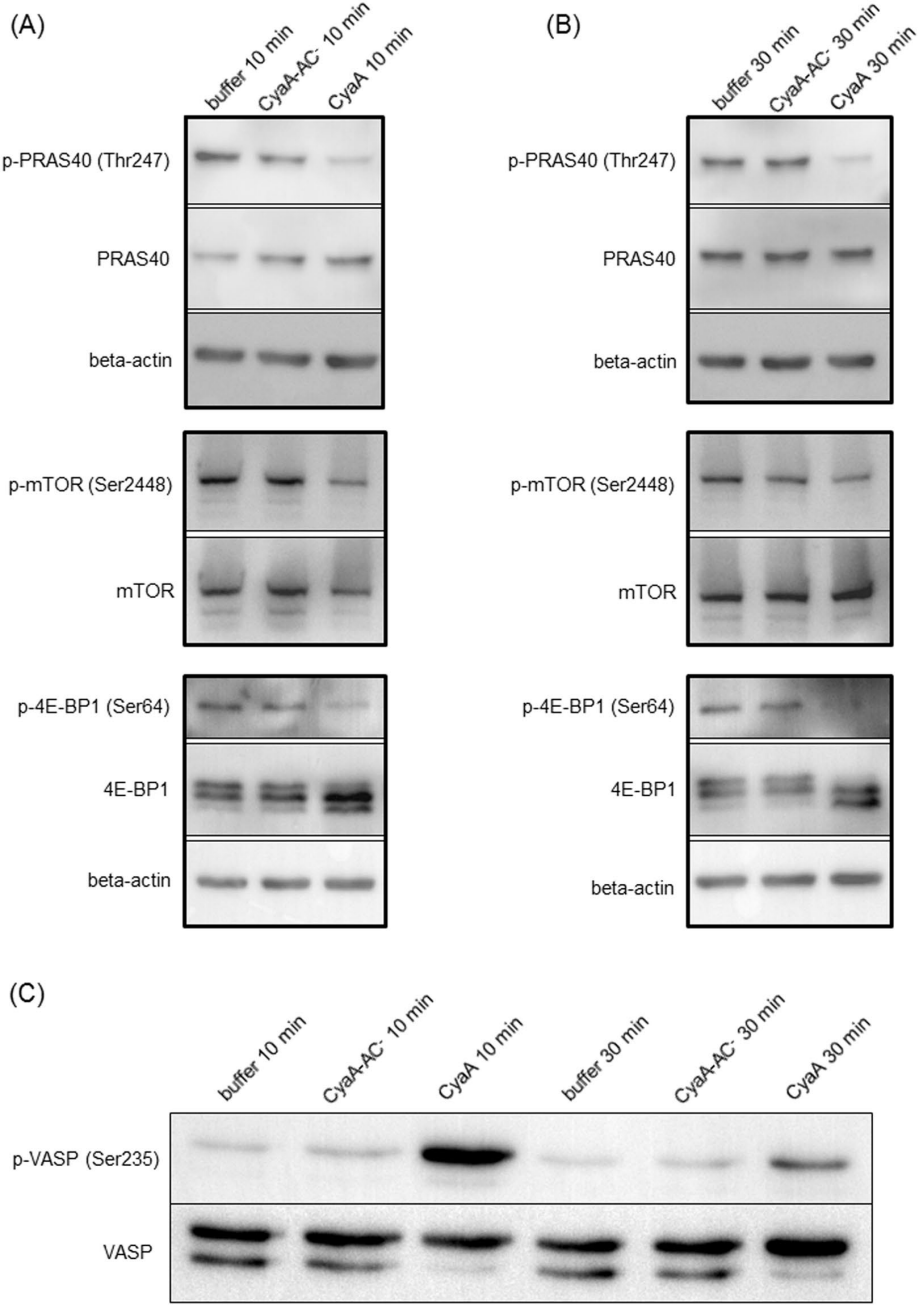
Changes in phosphorylation status of selected phosphoproteins. Members of mTOR signaling pathway after the toxin/toxoid treatment for (**A**) 10 minutes or (**B**) 30 minutes. (**C**) Phosphorylation status of serine 235 residue of cytoskeletal regulator VASP. In general, 10^6^ BMDCs in 1 mL were exposed for 10 or 30 minutes to 100 ng of CyaA or of the CyaA-AC^-^ toxoid, cellular lyzates were separated by SDS-PAGE and phosphorylation of indicated proteins was probed by Western blotting with specific antibodies. Images representative of at least three biological replicates are shown and quantifications of the Western blots of mTOR signaling pathway members are shown in Supplementary Fig. S6. Cropped images are shown and full-length blots are presented in Supplementary Fig. S7. “Buffer” stands for TUC-treated cells (TUC buffer −50 mM Tris-HCl, 8 M urea, 2 mM CaCl_2_, pH 8; used for toxin/toxoid dilution).

The impact of toxin and toxoid treatments on the phosphoproteome of BMDCs is summarized in Supplementary Fig. S3A-D. Only a small number of significantly regulated phosphosites was observed upon treatment of cells with the CyaA-AC^-^ toxoid for 10 minutes. Therefore, only samples from the 30 minutes time point for the toxoid were analyzed in more detail. When the significantly regulated phosphosites of the toxoid-treated sample were plotted against the matching phosphosites from the CyaA-treated condition, a prominent alteration of the phosphorylation state of the threonine residue 1465 (1462 in man) of tuberin (product of the Tsc2 gene) was observed (Fig. 2). Tuberin, a well described mTOR-signaling suppressor^45^, was dephosphorylated in CyaA-treated cells and was phosphorylated in toxoid-treated cells (Fig. 2A and B). cAMP signaling of CyaA thus inhibited the activity of the PI3K-Akt pathway accounting for tuberin phosphorylation^46^, while the permeabilization of cells by the toxoid activated this pathway. Besides, Western blot analysis revealed that phosphorylation of the PRAS40 protein, yet another mTOR signaling inhibitor^47^, was deregulated. PRAS40 exhibited diminished phosphorylation of the threonine residue 247^48^ in CyaA-treated cells (Fig. 3A,B, Supplementary Figs S6A and S7A-C). For mTOR itself, only a mild decrease in phosphorylation of serine residue 2448 was observed after 30 min of CyaA action on cells (Fig. 3B, Supplementary Figs S6B and S7D-E). To test if these changes affected the downstream targets of mTOR, the phosphorylation status of serine residue 64 of 4E-BP1 was probed by a specific antibody. Dephosphorylation at this site was detected both by SILAC phosphoproteomics and Western blotting (Fig. 3, Supplementary Figs S6C and D7F-H and Supplementary Table S1).

### CyaA-elicited phosphoproteome alterations modulate small GTPase signaling and actin cytoskeleton homeostasis

**Figure 4:**
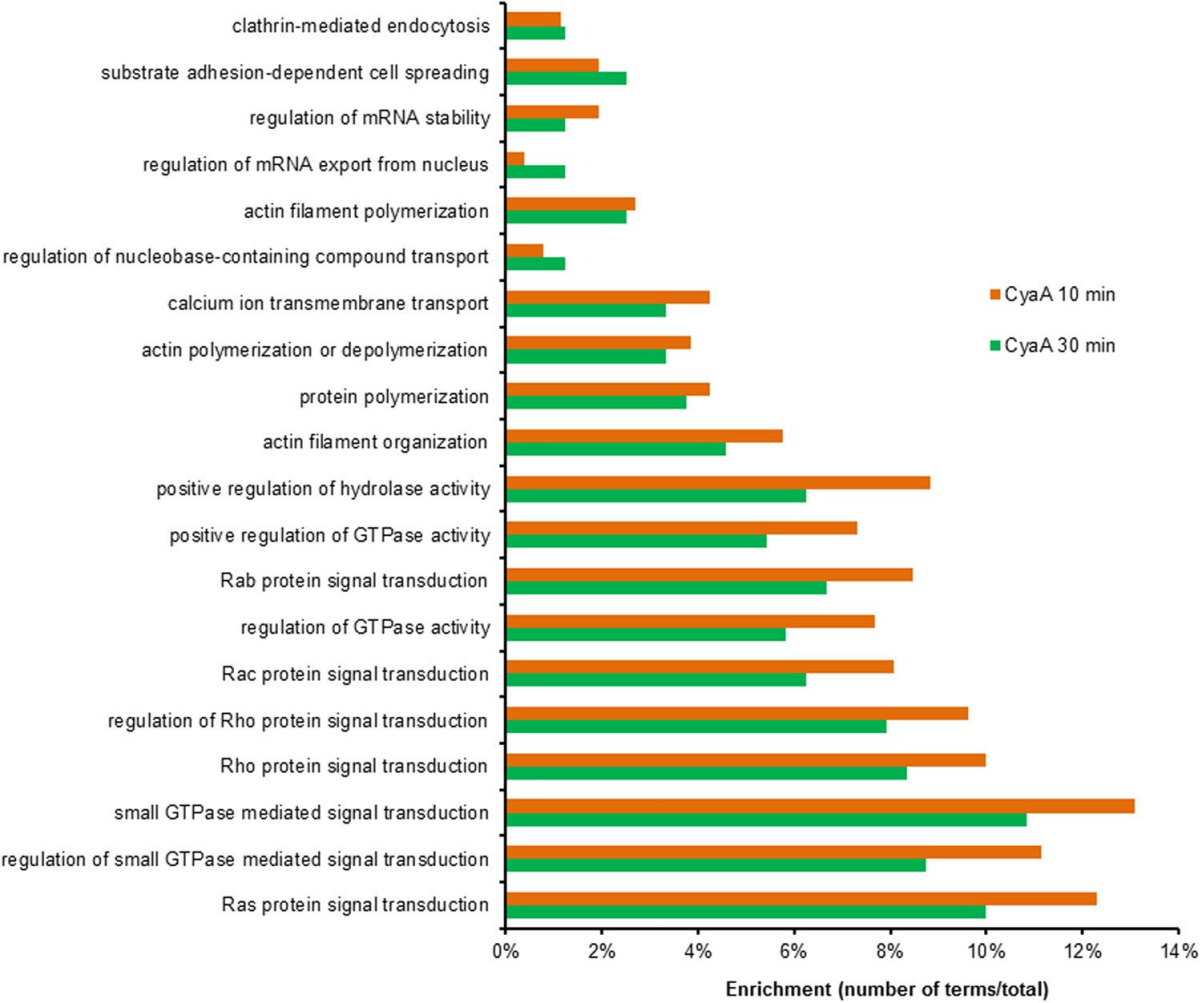
Functional analysis of CyaA-induced phosphoproteome changes. Analysis was performed using Gene Ontology Term enrichment and the top 20 terms (*p* ≤ 0.05) enriched in „Biological process“ library were ranked according to their p-values (corrected with Benjamini-Hochberg).The enrichment was counted as a number of genes belonging to each GO term divided by the total number of genes in each input gene set.

**Figure 5:**
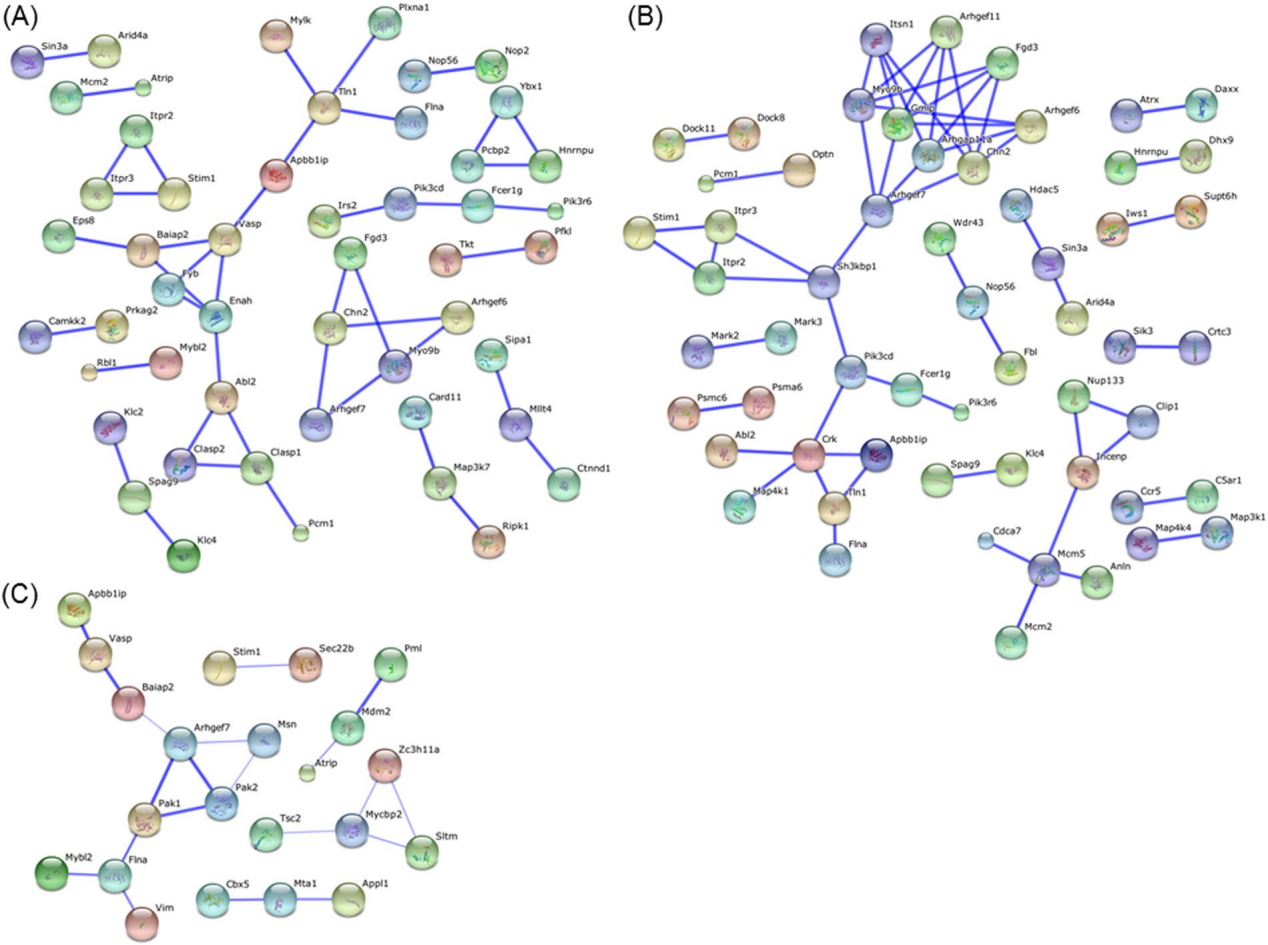
Functional association between significantly regulated phosphoproteins. Visualization of functional association of significantly regulated phosphoproteins was performed using STRING database v10 at the highest confidence level of 0.900 for (**A**) the sample treated with 100 ng/ml of WT CyaA for 10 min and (**B**) for 30 min. (**C**) Association at medium confidence 0.400 for significantly regulated phosphoproteins in the sample treated with 100 ng/ml CyaA-AC^-^ toxoid. C). The software-assigned coloring of nodes has no particular meaning and many of the indicated proteins are involved in several signaling pathways in parallel. Confidence view with stronger associations is represented by thicker lines.

When Gene Ontology (GO)^25^ terms enrichment analysis was applied to the phosphoproteome dataset, the action of CyaA on BMDCs resulted in the over-representation of terms related to GTPase signaling pathways and regulation of actin cytoskeleton (Fig. 4). This was further corroborated by construction of protein-protein interaction networks, using the STRING v10 database software^24^ to decode the signaling processes triggered by toxin action in BMDCs (Fig. 5A–C). The network capturing of CyaA action in the first 10 minutes consisted of 43 nodes of phosphoproteins (Fig. 5A), with the most highly interconnected nodes comprising Mena (Enah), Talin-1 (Tln1), VASP (Fig. 3C, Supplementary Fig. S7I-J) or Abl2 proteins, which are all involved in the regulation of cytoskeleton homeostasis^49–52^. This goes well with the previous observation that CyaA-elicited cAMP signaling provokes transient inhibition of RhoA and triggers massive actin cytoskeleton rearrangements and membrane ruffling^9^. CyaA-triggered signaling remained similar at 30 min of BMDC treatment with CyaA, yielding a network of 57 nodes (Fig. 5B). The interconnected cluster of the largest subnetwork then again contained components of the small GTPase signaling pathways, such as the small GTPase-activators Gmip^53^ and Arhgap11a^54^, which are involved in cytoskeleton homeostasis regulation. Altogether, these results show that the early effects of CyaA-elicited cAMP signaling largely affect small GTPases that regulate actin remodeling and cytoskeletal rearrangements.

For the sample treated with CyaA-AC^-^ (Fig. 5C), the STRING-based interaction network of affected phosphoproteins at the 30 min time point could only be constructed upon lowering of the stringency conditions (medium confidence 0.400). The resulting network then comprised, among others, the Heterochromatin protein 1 homolog alpha (CBX5), the E3 ubiquitin-protein ligase MDM2, the Rho guanine nucleotide exchange factor 7 (Arhgef7), PML and the SAFB-like transcription modulator (SLTM). Alterations of phosphorylation of these proteins might thus reflect the cell-permeabilizing activity of the CyaA-AC^-^ toxoid.

### CyaA-elicited cAMP signaling impacts on phosphorylation status of SIK kinases involved in immune response

**Figure 6:**
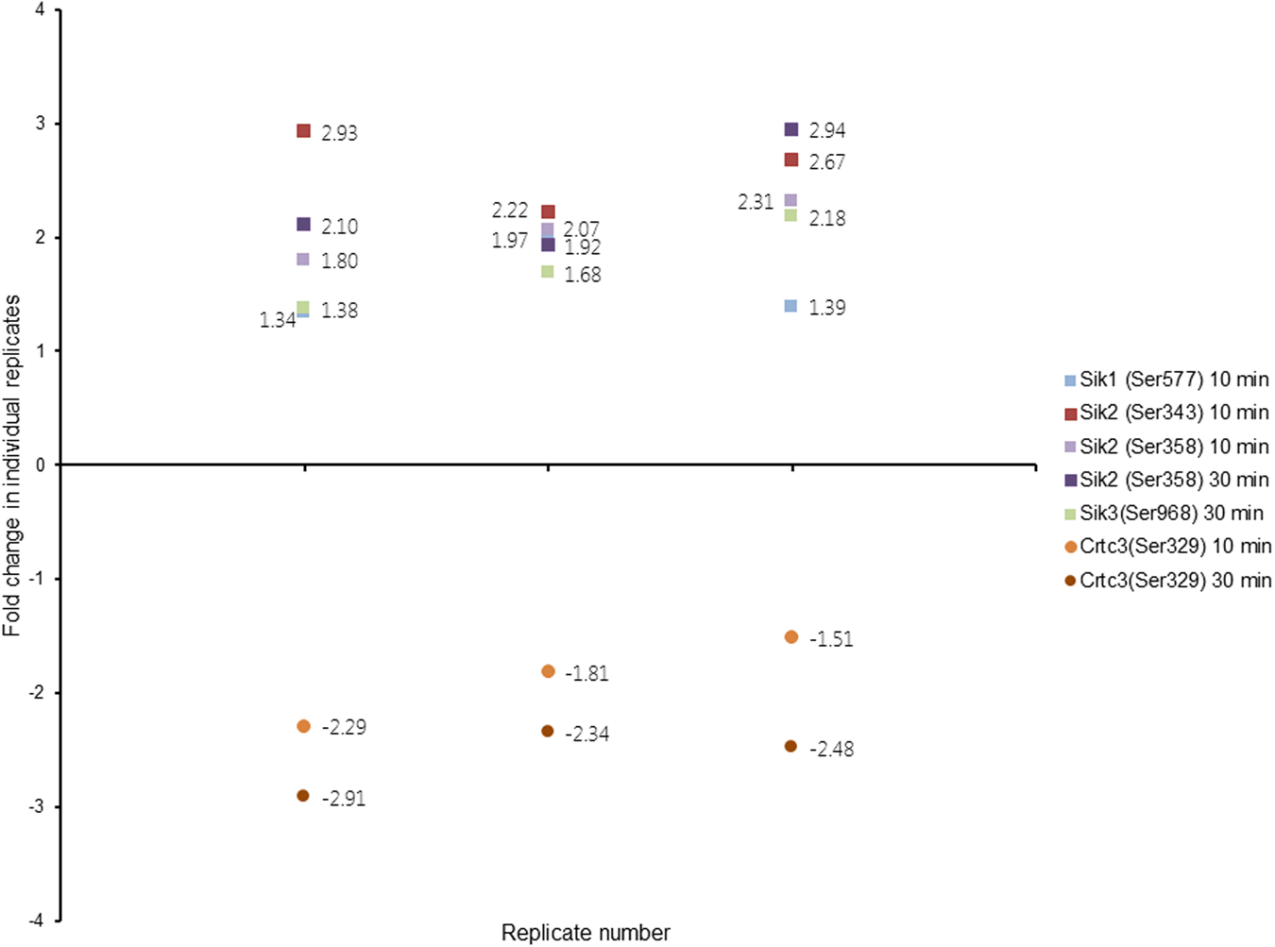
Changes in phosphorylation status of SIK family kinases and CRTC3/TORC3. Changes in the phosphorylation status of selected phosphosites are depicted here as fold change, representing the ratios of signals from toxin- or toxoid-treated samples to the corresponding buffer-treated control samples. Each data point represents one ratio value for one of the three replicates. The values > 0 represent phosphorylation, values < 0 represent dephosphorylation.

Based on their annotation^25,55,56^ (selected GO terms are listed in Supplementary Table S2) we identified 32 kinases that contain at least one phosphosite having a significantly altered phosphorylation state upon BMDCs treatment with CyaA (Supplementary Table S3). Among them, the phosphorylation of regulatory serine residues of several salt-inducible kinases (SIK), known for their involvement in modulation of immune responses^57–60^, was importantly altered (Fig. 6).

## Discussion

We report here the first phosphoproteome-wide analysis of the signaling impact of the cAMP-elevating activity of *B. pertussis* adenylate cyclase toxin in primary myeloid cells. The AC domain of the CyaA toxin binds calmodulin in the cytosol of cells and acquires an extremely high specific enzymatic activity of about 2,000 molecules of ATP converted to cAMP per second per AC enzyme molecule^61^. This yields a steep and uncontrolled elevation of intracellular cAMP concentration in cells. The data reported here thus provides important insight into the pleiotropic effects of cAMP signaling in myeloid cells and point to two main areas of CyaA toxin interference within normal DC physiology. These comprise cAMP signaling towards inhibition of mTOR activity, signaling promoting cytoskeletal rearrangements and signaling regulating cytokine production by dendritic cells, as discussed below.

**Figure 7:**
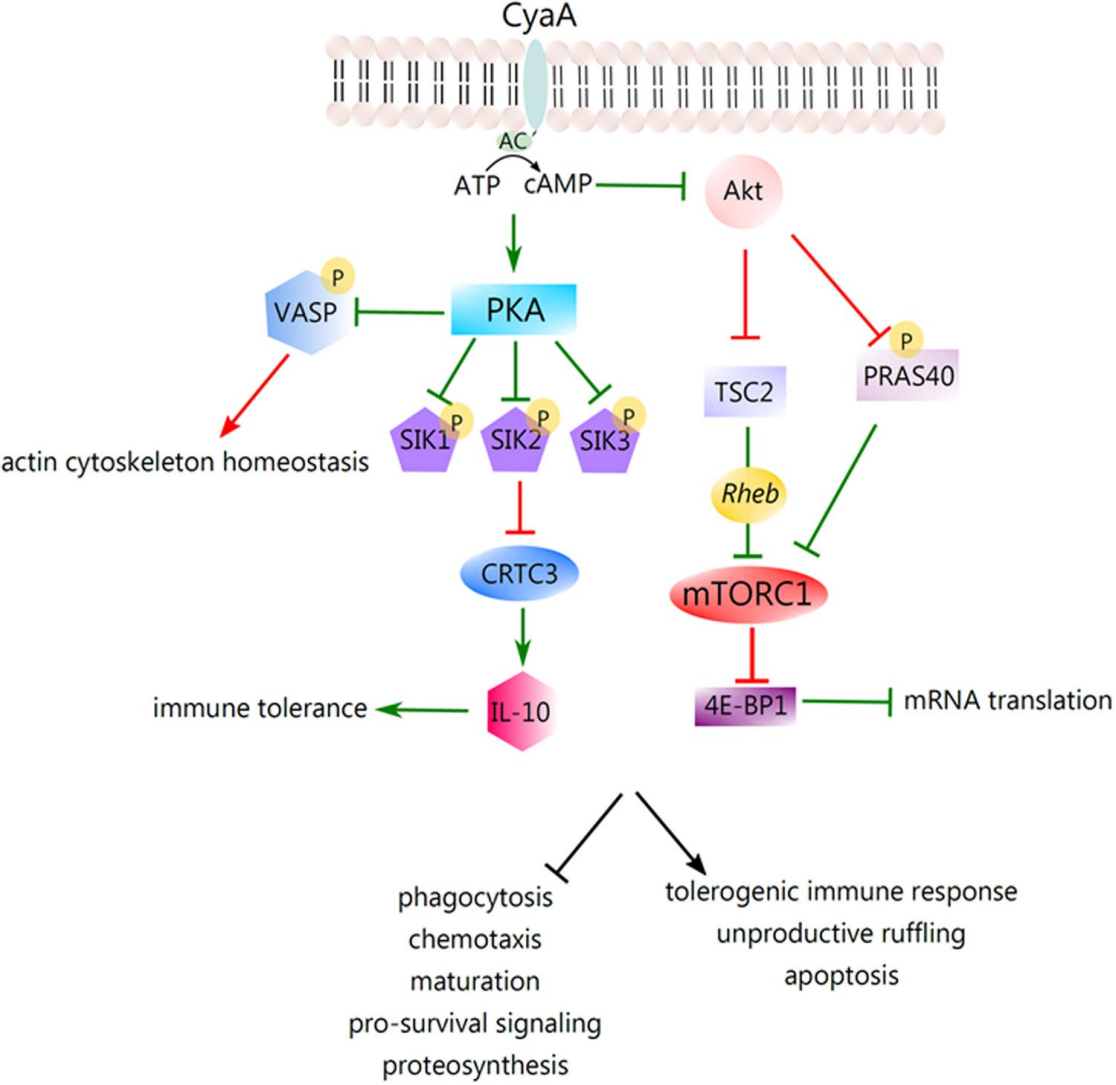
Schematic depiction of CyaA-induced signaling that would lead to induction of IL-10 production, inhibition of protein translation and deregulation of actin cytoskeleton homeostasis. Elevation of intracellular cAMP concentration due to the catalytic action of internalized AC domain of CyaA provokes activation of the protein kinase A, which performs an inhibitory phosphorylation of the SIK family proteins. This would promote nuclear localization of the transcriptional co-activator CRTC3. In parallel, PKA phosphorylates CREB and the phospho-CREB-CRTC3 complex would activate expression of the IL-10 gene. cAMP-dependent phosphorylation of VASP, presumably by PKA, affects actin cytoskeleton homeostasis. CyaA action further yields inhibition of Akt signaling, thus provoking inhibition of mTOR signaling. Pointed arrowheads indicate an activating effect and flat arrowheads indicate an inhibitory effect under normal physiological conditions. The red color of the arrowhead indicates an inhibitory effect, or interference, resulting from cAMP signaling elicited by the CyaA toxin and the green color indicates an enhancing effect of CyaA/cAMP signaling action. For the broader context of these signaling processes the reader is referred to dedicated reviews on immunity to *B. pertussis* infections and on CyaA toxin action^3,97^.

Pro-survival signaling of mTOR is negatively regulated by the protein tuberin, the activity of which is inhibited by phosphorylation of the threonine residue 1465 by Akt^45,46^. Tuberin is also known to be inhibited by Erk^62^. In contrast to previous reports using mouse or human macrophage or neutrophil cells^7,63^, we observed here that the Thr203 and Tyr205 residues in the activation loop of Erk1/2 were not dephosphorylated and the Erk kinases likely remained active even after 30 minutes of BMDC incubation with both the active CyaA toxin and its CyaA-AC^-^ toxoid (*c.f*. Supplementary Fig. S4). Nevertheless, we found by phosphoproteomics that in CyaA-treated cells the threonine 1465 residue of tuberin was dephosphorylated. However, we were unable to detect tuberin by immunoblotting with antibodies from various suppliers, which may suggest that only very low levels of tuberin are produced in BMDCs. Alternatively, these cells may express an isoform of tuberin that is not detected by the commercially available antibodies. Nevertheless, the dephosphorylation of threonine 1465 of tuberin, as detected by SILAC phosphoproteomics, would yield activated tuberin^46^ capable of inhibiting mTOR and its pro-survival signaling. In line with this, WT CyaA action caused inhibition of Akt, as observed by dephosphorylation of serine 475 and threonine 308 residues^64^ (see Supplementary Fig. S8 and the work of Ahmad *et al*.^7^). Deactivation of Akt kinase also yielded dephosphorylation of the threonine 247 residue of PRAS40, promoting mTOR inhibition^47^ (Figs 3 and 7). As a result, the downstream effectors of mTOR involved in the synthesis of cellular proteins, such as 4E-BP1 (serine 64)^65^ (c.f. Fig. 3 and Supplementary Fig. S7F-H) and p70S6K (serine 427)^66^, were found to be dephosphorylated in CyaA-treated cells (Supplementary Table S1), where hypophosphorylated 4E-BP1 strongly binds to eukaryotic translation initiation factor and prevents translation^67^. The proposed model of mTOR signaling regulation by CyaA in BMDCs is then shown in Fig. 7. mTOR acts as an important regulator of the antibacterial inflammatory response in monocytes, macrophages and primary dendritic cells^68,69^. Inhibition of mTOR also leads to the activation and nuclear translocation of NF-κB and CyaA-provoked inhibition of mTOR would go well with our previous observation that NF-κB is activated upon CyaA-produced elevation of cAMP in macrophages^6,68^. The CyaA-provoked inhibition of mTOR is likely to have rather pleiotropic effects and these would vary in different DC subtypes^70,71^. For example, inhibition of mTOR was reported to result in the inhibition of IL-4-dependent maturation of BMDCs and in lowering of their T cell stimulatory capacity^72,73^. This would go well with our observations that CyaA treatment skews TLR-induced maturation of dendritic cells, inhibits their capacity to present antigens to both CD4^+^ and CD8^+^ T cells and enables the toxin-treated DCs to expand regulatory T cells *in vitro* ^74^.

CyaA action elicited phosphorylation of the hub-like Crk adaptor protein on the conserved serine 125 residue that is located between the SH2 and SH3 domains (c.f. Supplementary Table S1). This is likely to be of relevance during *Bordetella* infections, as hijacking of Crk appears to be implicated in bacterial pathogenesis through diverse mechanisms, including subversion of cellular ruffle formation (reviewed in Martinez-Quiles *et al*.^75^). The Crk-associated proteins retained in the interaction network (c.f. Fig. 5B) then comprised Talin-1, Filamin-A and the APBB1-interacting protein 1/RIAM, which are involved in cellular actin remodeling and phagocytosis. It is of note that CyaA action was previously reported to manipulate RhoA signaling and induce unproductive membrane ruffling^9^. Phosphorylation of the vasodilator-stimulated phosphoprotein (VASP) by the cAMP-activated PKA was then shown to play an important role in membrane ruffle formation, causing a significant reduction of the retraction of membrane ruffles^76^. In line with that, increased phosphorylation of the known PKA/PKG target VASP on serine residue 235 was observed in WT CyaA-treated BMDCs samples (c.f. Fig. 3C and Supplementary Table S1). This VASP site lies within the EVH2 domain that has been reported to account for VASP oligomerization and F-actin binding^77^. Phosphorylation of VASP then prevents actin polymerization by inhibiting the formation of profilin-actin complexes in myogenic cells^50^. This modification may thus complement the impact exerted on cytoskeleton rearrangements by CyaA-provoked RhoA inhibition. However, what causes this particular RhoA inhibition remains unknown.

Indeed, three possible mechanisms of small GTPase inhibition due to cAMP signaling can plausibly be invoked. The activities of small GTPases like RhoA, Cdc42 or Rac1 involve cycling between GDP-bound (inactive) and GTP-bound (active) states and are regulated by the interplay of activating GEFs (activating guanine nucleotide exchange factor) and inhibitory GAPs (GTPase-activating proteins)^78^. Besides, RhoA, as other small GTPases, can also be kept in an inactive state by guanine nucleotide dissociation inhibitors (GDIs)^79^. We observed that the action of CyaA provoked alterations of the phosphorylation state of several members of the GEF protein family, some of which were previously shown to directly promote RhoA activity^80^. For example, the protein known as Solo (or ARHGEF40) was shown to specifically induce RhoA activity in vascular endothelial cells^81^. Here, the treatment of BMDCs with CyaA resulted in dephosphorylation of the serine residue located in the region closest to the GEF domain of Solo. Another RhoA activator that is regulated by CyaA action is PDZRhoGEF (PRG; also known as ARHGEF11). This protein was found to be phosphorylated on the serine residue (c.f. Supplementary Table S1) that is localized in the center of the proposed key autoinhibitory element^82^, which may affect the conformation of the surrounding regions and may eventually lead to changes in PRG and affect its affinity for RhoA.

Among the GAPs known to be involved in RhoA regulation, three were found to be phosphorylated upon CyaA action on BMDCs (c.f. Supplementary Table S1), including the unconventional myosin-IXb (*Myo9b*), whose depletion was shown to impair the migratory capacity of dendritic cells and alter their interaction with T cells^83^. The other GAPs found to be regulated upon CyaA action was the ARHGAP18 protein, which is required for cellular polarization involved in cell migration^84^ and the ARHGAP11A protein, a key regulator of RhoA during cell division (also called MP-GAP or M Phase GAP)^85^.

The prolonged course of the whooping cough disease, often lasting for up to 12 weeks or longer, raises the hypothesis that *B. pertussis* infection may exert long-lasting effects on the cells of host mucosa and these would persist long after the bacteria have been cleared from the airway^86,87^. Although this effect is presumably due to the changes in epithelial cells, we hypothesize, that also airway intraepithelial DC might be affected similarly. Therefore, we examined the phosphorylation state of epigenetic effectors and chromatin remodeling proteins^88^ in CyaA-treated DC. Indeed, the class II histone deacetylase, HDAC5, was found to be dephosphorylated on serine 270 (serine 279 in human orthologue) in the nuclear localization signal. This would go well with the report of Taniguchi *et al*.^89^ that cAMP-stimulated nuclear import of HDAC5 requires dephosphorylation of Ser 270 by protein phosphatase 2 A (PP2A). Furthermore, CyaA action on DC provoked alterations of phosphorylation state of additional proteins that are involved in chromatin remodeling and epigenetic regulation. Among them, the *Cbx5* gene-encoded HP1α protein, involved in propagation and maintenance of pericentric heterochromatin^90^, was found to be phosphorylated on serine 93. Another Chromo domain-containing protein that was significantly phosphorylated (on serine 305) upon CyaA treatment of BMDC was the transcriptional repressor CBX8, also known as PC3^91^. In addition, significant phosphorylation of the replication-dependent histone H1.4 (*Hist1h1e*) on threonine 35 and serine 36 was observed. This goes well with the observation that H1.4 can be phosphorylated by PKA during mitosis and when it dissociates from mitotic chromatin^92^. CyaA-triggered changes in chromatin state and histone modifications could then also result in alteration of pre-mRNA splicing^93^ and numerous other effects on gene expression and cell cycle progression.

An important effect of CyaA toxin action, with relevance for host defense capacities, was observed for the phosphorylation state of the regulatory serine residues of the salt-inducible kinases (SIK). These are known for their involvement in modulation of immune responses^57–60^. Indeed, inhibition of SIKs by cAMP-activated and PKA-mediated phosphorylation was shown to suppress secretion of the pro-inflammatory cytokines IL-6, IL-12, and TNF-α by macrophages and appears to promote formation of regulatory (M2b) macrophages^59^. One of the SIK targets is the CREB-regulated transcription co-activator 3 (CRTC3) that can be phosphorylated at several serine residues. SIK-mediated phosphorylation was then shown to promote sequestering of CRTC3 into cytosolic complexes with the 14-3-3 protein, inhibiting CRTC3-assisted transcription of CREB-dependent genes, such as the anti-inflammatory cytokine IL-10^59^. In turn, phosphorylation of CRTC3 is inhibited by the cAMP-activated PKA and this enables translocation of the non-phosphorylated CRTC3 into the nucleus, where interaction of CRTC3 with CREB, activated by CyaA-produced cAMP^94^, may upregulate IL-10 gene transcription. In line with that we observed significant dephosphorylation of the serine 329 residue of CRTC3 and increased phosphorylation of the upstream regulatory kinases SIK1 (SIK), SIK2 (QIK) and SIK3 (QSK) in CyaA-treated BMDCs (*c.f*. Fig. 6). There was also enhanced phosphorylation of SIK1 on serine 577, which would promote the export of SIK1 from cell nucleus into the cytoplasm^95^, rescuing CRTC3-dependent transcription. Similarly, in CyaA-treated cells an increased phosphorylation of serine 343 at the critical regulatory site of SIK2/QIK was observed, where this modification was shown to inhibit the ability of SIK2/QIK to phosphorylate CRTC3^60^. Moreover, we also observed the enhanced phosphorylation of serine 358 of SIK2. This modification is known to promote cytoplasmic localization of SIK2 into complexes that contain the 14-3-3 proteins^96^. Last but not least, SIK3 phosphorylation was also observed. All of these results are thus in line with previous reports that TLR-activated DCs exposed to CyaA secrete increased amounts of the IL-10 cytokine and promote IL-10 secretion by regulatory T cells^74^. The sum of the data then allows proposing a model of immunosubversive signaling activated by CyaA in myeloid cells, as depicted in Fig. 7. It predicts that activation of PKA isoforms by the CyaA-produced cAMP yields inhibitory phosphorylation of the SIK family kinases and CRTC3-activated transcription of the CREB-dependent IL-10 gene. Altogether these results document the pleiotropic immunosubversive effects of the massive cAMP signaling that is elicited in primary myeloid cells by the AC enzyme action of the CyaA toxin.

## Electronic supplementary material

Supplementary Info

Supplementary dataset
