# Supplementary material for: Phosphoproteomics of cAMP signaling of Bordetella adenylate cyclase toxin in mouse dendritic cells
Source: Sci Rep. 2017 Nov 24;7:16298. doi: 10.1038/s41598-017-14501-x (PMC5701129; doi:10.1038/s41598-017-14501-x)
Supplement: Supplementary file 1 — Supplementary Info [file 41598_2017_14501_MOESM1_ESM.pdf]

Supplementary Information

Phosphoproteomics of cAMP signaling of *Bordetella* adenylate cyclase toxin in mouse dendritic cells

Jakub Novák[1], Ivo Fabrik[2], Irena Linhartová[1], Marek Link[2], Ondřej Černý[1], Jiří Stulík[2] and Peter Šebo[1*]

[1] Institute of Microbiology of the CAS, v.v.i., Prague, Czech Republic; [2] Department of Molecular Pathology and Biology, Faculty of Military Health Sciences in Hradec Kralove, University of Defence in Brno, Czech Republic

Supplementary Methods:

Generation and SILAC labelling of bone marrow-derived DCs (BMDCs)

BMDCs were generated from bone marrow progenitors isolated from femurs and tibias of 6- to 8-week-old female C57BL/6 mice. Approximately 1 × 10^7 bone marrow cells were seeded on 10 cm tissue plastic Petri dish into 10 mL of RPMI-1640 media containing 10% (v/v) fetal bovine serum (FBS; Sigma Aldrich) and penicillin/streptomycin. Cells were incubated at 37 °C in a humidified atmosphere of 5% CO_2 overnight. Non-adherent cells were collected and seeded into fresh dishes in RPMI-1640 with 10% FBS and 5% (v/v) of supernatant from Ag8653 cells transfected by cDNA for production of murine GM-CSF. BMDCs were passaged every 2−3 days and suspensions were harvested on the day 9 of cultivation. Routinely, ~80% of the population was $CD11c^+$ (not shown).

For SILAC experiments, BMDCs were metabolically labelled according to previously described protocol[1]. SILAC labelling medium was prepared by the addition of ^{13}C-labelled L-arginine [$^{13}C_6$] and L-lysine [$^{13}C_6$] (final concentrations 0.4 mM and 0.8 mM, respectively) and by the addition of unlabeled L-proline (final concentration 2.6 mM) to SILAC RPMI-1640 (Sigma Aldrich). Concentration of dialyzed FBS (Sigma Aldrich) in the medium during the SILAC-labelling cultivation was 10% (v/v).

Protein digestion, sodium deoxycholate (SDC) removal and peptide desalting

Proteins were reduced by the addition of dithiotreitol (DTT; final concentration 10 mM) for 1 h at 37 °C followed by the alkylation with iodoacetamide (IAA; final

concentration 20 mM) for 30 min at RT in the dark. The excess of IAA was quenched by the addition of DTT to a final concentration of 20 mM and the reaction was left to proceed for 15 min at RT. Proteins were digested by trypsin (Promega) at a ratio 50:1 (w/w) at 37 °C overnight. Digestion was stopped by the addition of TFA to a final concentration of 1% (v/v) to precipitate SDC. Suspensions were mixed with an equal volume of ethyl acetate, vortexed and centrifuged. Upper organic phase was removed and the extraction process was repeated four times to completely extract SDC. Water phase containing peptides was then desalted on Discovery DSC-18 SPE cartridges (500 mg/3 mL; Sigma Aldrich) and the eluate in 80% ACN/0.1% TFA was vacuum dried.

Fractionation of peptides by hydrophilic interaction chromatography (HILIC)

3 to 5 mg of peptide material was injected onto TSKgel Amide-80 HR column (5 µm, 4.6 × 250 mm) with guard column (5 µm, 4.6 × 10 mm; both Tosoh Bioscience) using Alliance 2695 liquid chromatograph (Waters) under conditions of 20% mobile phase A (2% ACN/0.1% TFA) and 80% mobile phase B (98% ACN/0.1% TFA) at a flow rate of 0.5 mL/min. Peptide separation was performed by a linear gradient formed by mobile phase A and mobile phase B, from 80 to 60% of mobile phase B in 40 min and from 60 to 0% of mobile phase B in 5 min. Column temperature was kept constant at 30 °C, and the separation was monitored at 215 nm.

Liquid chromatography-mass spectrometry (LC-MS)

The Ultimate 3000 RSLCnano system connected with a Q Exactive mass spectrometer (Thermo Scientific) were used for instrumental analysis. Approximately one third of the sample material from each phosphopeptide fraction was introduced onto a trap column (PepMap100 C18, 3 µm, 0.075×20 mm) and peptides were separated by running a linear gradient (0.1% FA in water as phase A; 80% acetonitrile, 20% water and 0.1% FA as phase B) from 4 to 34% B in 48 min and from 34 to 55% B in 10 min, at a flow rate of 300 nL/min, using an analytical column (PepMap C18, 2 µm, 0.075×150 mm). The full MS/Top10 setup was used for mass spectra acquisition. The positive ion MS spectra from 350-1750 *m/z* range were obtained in the Orbitrap at a resolution of 70,000 (at *m/z* 200). Multiply charged precursor ions with minimal threshold intensity of 5×10^4 counts and not fragmented during previous 30s were admitted for higher energy collisional dissociation (HCD).

Tandem mass spectra were acquired with following settings; resolution at 17,500, AGC target value at 1×10^5, maximum ion injection time at 100 ms, and normalized collision energy set to 27. Data acquisition was under control of Xcalibur software v3.0.

Phosphosite identification and quantification

MaxQuant ver. 1.5.1.0 coupled with Andromeda search engine[2,3] was used for phosphosite identification and quantification. The obtained data were searched against FASTA database consisting of reference proteome for *Mus musculus* downloaded from Uniprot (UP000000589; May 7, 2015) and the sequences of wild type and toxoid versions of CyaA (see supplementary txt file). False discovery rate (FDR) estimation of peptide identification was based on target-decoy approach, using reverted search database (with swap of lysine and arginine with neighbouring amino acid) as a decoy. Parameters of MaxQuant search were: mass tolerance for the first search 20 ppm, for the second search from recalibrated spectra 4.5 ppm (with individual mass error filtering enabled); maximum of 2 missed cleavages; maximal charge per peptide z = 7; minimal length of peptide 7 amino acids, maximal mass of peptide 4600 Da; carbamidomethylation (C) as fixed and phosphorylation (STY), oxidation (M), and acetylation (protein N-term) as variable modifications with the maximum number of variable modifications per peptide set to 5. Trypsin with no cleavage restriction was set as a protease. Mass tolerance for fragments in MS/MS was 20 ppm, taking the 12 most intensive peaks per 100 Da for search (with enabled possibility of co-fragmented peptide identification). Minimal Andromeda score for modified peptides was 40 and minimal delta score for modified peptides was 6. FDR filtering on peptide spectrum match was 0.01 with separate FDR filtering for each modification set to 0.01. For peptide quantitation, Arg+6 [$^{13}C_6$] and Lys+6 [$^{13}C_6$] were set as labels in heavy channel (or in light channel for label-swap experiments to obtain inverted H/L ratios) with re-quantify function enabled. Ratios for individual phosphosites were derived from normalized ratios of the least modified phosphopeptides in a given replicate.

Bioinformatic analyzes

- ***Significance analysis by Global Mean Rank Test (GMRT)*** - First, phosphosites classified as contaminants from MaxQuant search (based on the

implemented database of contaminants in software) were filtered out. Significantly regulated phosphosites for given experimental condition (time point and toxin-/toxoid-treatment, Figure S1) were then found by GMRT using R package MeanRankTest (https://www.evotec.com/MeanRankTest) with parametric FDR level set to 0.05. Only those phosphosites having control/treated ratios in all three biological replicates of the respective experimental condition were considered for testing.

- ***Construction of Phosphorylation motifs by motif-X*** – To assemble significantly enriched phosphorylation motifs the web-based motif-X algorithm[4] was used with IPI mouse proteome as a search background. Significance for a positive hit was set to 0.000001. Fold increase of extracted motifs, used as the indicator of the enrichment level, was calculated as follows: (foreground matches/foreground size) / (background matches/background size).
- ***Kinase motif enrichment analysis by Fisher exact test*** – Only phosphosites identified with localization probability >0.75, and quantified in all three replicates for the corresponding experimental condition, were considered. For these, potential kinases were found based on the Human Protein Reference Database (Release 9; http://www.hprd.org/) kinase motif atlas implemented in Perseus software ver. 1.5.1.6 (http://www.perseus-framework.org/). Fisher exact test was then used to find enriched kinase motifs in groups of significantly regulated phosphosites per given experimental condition (Benjamini-Hochberg FDR level set to 0.05). Enrichment factors for individual kinase motifs were counted as follows: (number of hits for particular kinase / number of significantly regulated phosphosites) / (number of hits particular kinase in the whole given cluster / total size of the dataset).
- ***Analysis of GO enrichment by:***
  - ***ClueGO plugin[5]*** – Parameters of enrichment test were as follows: GO Term interval 3 – 8, minimum of 3 genes/3 % of all genes from the input. Two-sided hypergeometric test with Benjamini-Hochberg correction was performed, GO Term fusion was allowed and pV for found terms was set to be higher than 0.05.

Western blots

Cell lysis was performed in RIPA buffer (with 1mM EDTA, PhosSTOP and cOmplete Mini, EDTA-free Protease Inhibitor Cocktail (Roche)). Lysates from cells were then separated by SDS-PAGE and transferred to PVDF membrane - 5% gel and wet transfer without methanol or SDS were used for mTOR analysis, 7.5% gel and semi-dry transfer were then used for analyzes of other proteins. Membranes were decorated with appropriate antibodies (dilution 1:5000 for β-Actin, 1:1000 for other antibodies) and visualized using Enhanced chemiluminescence (ECL) with SuperSignal West Femto Maximum Sensitivity Substrate (Thermo Scientific). Chemiluminesce was measured using G:BOX Chemi XRQ (Syngene) with Synoptics 4.0MP camera and GeneSys image aquisition software (version 1.5.2.0, database version 1.85). Densitometric analysis was performed using ImageJ[6] software distribution Fiji[7] (version 1.51n).

List of antibodies

Product name	Cat. #	Manufacturer	Ref.
4E-BP1 (53H11) Rabbit mAb	9644	CST	[8]
Phospho-4E-BP1 (Ser65) Antibody	9451	CST	[9]
Akt (pan) (40D4) Mouse mAb	2920	CST	[10]
Phospho-Akt (Ser473) Antibody	9271	CST	[9]
Phospho-Akt (Thr308) (C31E5E) Rabbit mAb	2965	CST	[11]
p44/42 MAPK (Erk1/2) (137F5) Rabbit mAb	4695	CST	[12]
Phospho-p44/42 MAPK (Erk1/2) (Thr202/Tyr204) (D13.14.4E) XP® Rabbit mAb	4370	CST	[12]
β-Actin (8H10D10) Mouse mAb	3700	CST	[13]
mTOR (7C10) Rabbit mAb	2983	CST	[14]
Phospho-mTOR (Ser2448) (D9C2) XP® Rabbit mAb	5536	CST	[15]
PRAS40 (D23C7) XP® Rabbit mAb	2691	CST	[8]
Phospho-PRAS40 (Thr246) (C77D7) Rabbit mAb	2997	CST	[8]
VASP (9A2) Rabbit mAb	3132	CST	[16]
Phospho-VASP (Ser239) Antibody	3114	CST	[17]

References:

1 Fabrik, I. *et al.* Application of SILAC labeling to primary bone marrow-derived dendritic cells reveals extensive GM-CSF-dependent arginine metabolism. *J Proteome Res* 13, 752-762, doi:10.1021/pr4007798 (2014).

2 Cox, J. *et al.* Andromeda: a peptide search engine integrated into the MaxQuant environment. *J Proteome Res* 10, 1794-1805, doi:10.1021/pr101065j (2011).

3 Cox, J. & Mann, M. MaxQuant enables high peptide identification rates, individualized p.p.b.-range mass accuracies and proteome-wide protein quantification. *Nat Biotechnol* 26, 1367-1372, doi:10.1038/nbt.1511 (2008).

4 Chou, M. F. & Schwartz, D. Biological sequence motif discovery using motif-x. *Curr Protoc Bioinformatics* Chapter 13, Unit 13.15-24, doi:10.1002/0471250953.bi1315s35 (2011).

5 Bindea, G. *et al.* ClueGO: a Cytoscape plug-in to decipher functionally grouped gene ontology and pathway annotation networks. *Bioinformatics* 25, 1091-1093, doi:10.1093/bioinformatics/btp101 (2009).

6 Schneider, C. A., Rasband, W. S. & Eliceiri, K. W. NIH Image to ImageJ: 25 years of image analysis. *Nat Methods* 9, 671-675 (2012).

7 Schindelin, J. *et al.* Fiji: an open-source platform for biological-image analysis. *Nat Methods* 9, 676-682, doi:10.1038/nmeth.2019 (2012).

8 Merhi, A., Delrée, P. & Marini, A. M. The metabolic waste ammonium regulates mTORC2 and mTORC1 signaling. *Sci Rep* 7, 44602, doi:10.1038/srep44602 (2017).

9 Poburski, D. *et al.* Insulin-IGF signaling affects cell transformation in the BALB/c 3T3 cell model. *Sci Rep* 6, 37120, doi:10.1038/srep37120 (2016).

10 Panigrahi, S. K., Manterola, M. & Wolgemuth, D. J. Meiotic failure in cyclin A1-deficient mouse spermatocytes triggers apoptosis through intrinsic and extrinsic signaling pathways and 14-3-3 proteins. *PLoS One* 12, e0173926, doi:10.1371/journal.pone.0173926 (2017).

11 Li, X. *et al.* miR-18a counteracts AKT and ERK activation to inhibit the proliferation of pancreatic progenitor cells. *Sci Rep* 7, 45002, doi:10.1038/srep45002 (2017).

12 Lee, H. *et al.* Targeted disruption of TC-PTP in the proliferative compartment augments STAT3 and AKT signaling and skin tumor development. *Sci Rep* 7, 45077, doi:10.1038/srep45077 (2017).

13 Yang, F. *et al.* Epigenetic modifications of interleukin-6 in synovial fibroblasts from osteoarthritis patients. *Sci Rep* 7, 43592, doi:10.1038/srep43592 (2017).

14 Mu, Y. *et al.* Diet-induced obesity impairs spermatogenesis: a potential role for autophagy. *Sci Rep* 7, 43475, doi:10.1038/srep43475 (2017).

15 Sakamoto, S., Miyara, M., Sanoh, S., Ohta, S. & Kotake, Y. Mild MPP(+) exposure-induced glucose starvation enhances autophagosome synthesis and impairs its degradation. *Sci Rep* 7, 46668, doi:10.1038/srep46668 (2017).

16 Sakai, M. *et al.* The GCN5-CITED2-PKA signalling module controls hepatic glucose metabolism through a cAMP-induced substrate switch. *Nat Commun* 7, 13147, doi:10.1038/ncomms13147 (2016).

17 Döppler, H. R., Bastea, L. I., Lewis-Tuffin, L. J., Anastasiadis, P. Z. & Storz, P. Protein kinase D1-mediated phosphorylations regulate vasodilator-stimulated phosphoprotein (VASP) localization and cell migration. *J Biol Chem* 288, 24382-24393, doi:10.1074/jbc.M113.474676 (2013).

Supplementary Tables Legends:

Supplementary Table S1. Phosphosites quantified in all three replicates in at least one sample

Explanation of column headers of Supplementary Table S1:

**Ratio H/L normalized**	Normalized ratios of heavy (control) vs. Light (treated) signal for particular phosphosite. For label-swap experiments (signed with "LS") are these values inverted. Rep1-3 indicates replicate number.
**Intensity**	Sum of all intensities (all signals from both light and heavy peptides) in particular replicate. Provides information about the strength of the signal.
**Intensity log**	Logaritmized (log10) Intensity value
**Localization prob**	Probability of correctly localized de/phosphorylation in particular peptide. Values > 0.75 are considered as correctly localized.
**Ratio H/L nmods**	Value indicating how many de/phosphorylations contained peptide, which was used for quantification of particular phosphosite.
**Number of Phospho (STY)**	Number of de/phosphorylations observed for peptide containing particular phosphosite.
**WT-10m-significant**	"+" sign indicates significantly regulated phosphosites according to MeanRank test (FDR = 0.05). Only phosphosites which were quantified in all three replicates were used.
**WT-10m-q**	Q value of particular phosphosite in the sample according to MeanRank test. Used to determine cut-off FDR 0.05, only phosphosites with q < 0.05 were considered significantly regulated.
**Proteins**	All possible proteins containing particular phosphosite.
**Leading proteins**	Proteins with the best coverage by identified peptides.
**Protein**	One chosen protein from the "Leading proteins" group.
**Protein names**	Name/s of the proteins bearing particular phosphosite.
**Gene names**	Gene name/s of the proteins bearing particular phosphosite.
**Fasta headers**	FASTA header/s of the protein/s containing particular phosphosite.
**Amino acid**	De/phosphorylated amino acid.
**Positions within proteins**	Phosphosite position/s in protein/s.
**Positions**	Phosphosite position/s in protein/s restricted only to proteins from "Leading proteins" group.
**Position**	Phosphosite position in protein from "Protein" column.
**Sequence**	Sequence containing particular phosphosite, including 15 amino

**window**	acids up and downstream.
**Phospho (STY) Probabilities**	Highlighted position of de/phosphorylation in peptide and its localization probability.
**id**	Unique identifier or every phosphosite.

Supplementary Table S2. GO terms linked to kinases and/or kinase activity, which were found annotated to proteins in our dataset

Supplementary Table S3. List of all significantly regulated kinases

Supplementary Tables:

Supplementary Table S2. GO terms linked to kinases and/or kinase activity, which were found annotated to proteins in our dataset

Ontology	Molecular Function
**Name**	**ID**
1-phosphatidylinositol-3-kinase activity	[GO:0016303]
1-phosphatidylinositol-4-phosphate 3-kinase activity	[GO:0035005]
6-phosphofructokinase activity	[GO:0003872]
AMP-activated protein kinase activity	[GO:0004679]
calmodulin-dependent protein kinase activity	[GO:0004683]
creatine kinase activity	[GO:0004111]
cyclin-dependent protein serine/threonine kinase activity	[GO:0004693]
diacylglycerol kinase activity	[GO:0004143]
diphosphoinositol-pentakisphosphate kinase activity	[GO:0033857]
histone kinase activity	[GO:0035173]
inositol hexakisphosphate 1-kinase activity	[GO:0052723]
inositol hexakisphosphate 3-kinase activity	[GO:0052724]
inositol hexakisphosphate 5-kinase activity	[GO:0000832]
inositol-1,3,4,5,6-pentakisphosphate kinase activity	[GO:0000827]
JUN kinase kinase kinase activity	[GO:0004706]
kinase activity	[GO:0016301]
lipid kinase activity	[GO:0001727]
MAP kinase kinase activity	[GO:0004708]
MAP kinase kinase kinase kinase activity	[GO:0008349]
myosin light chain kinase activity	[GO:0004687]
non-membrane spanning protein tyrosine kinase activity	[GO:0004715]
phosphatidylinositol-4,5-bisphosphate 3-kinase activity	[GO:0046934]
protein serine/threonine kinase activity	[GO:0004674]
protein tyrosine kinase activity	[GO:0004713]
ribosomal protein S6 kinase activity	[GO:0004711]
RNA polymerase II carboxy-terminal domain kinase activity	[GO:0008353]
tau-protein kinase activity	[GO:0050321]

Supplementary Table S3. List of all significantly regulated kinases

**Gene name**
Araf
Aak1
Abl2
Baz1b
Cad
Camkk2
Cdk13
Cdk18
Clk3
Dapk2
Dgkz
Lmtk2
Map3k1
Map3k7
Map4k1
Map4k4
Mark2
Mark3
Mink1
Mylk
Pfkl
Pik3cd
Ppip5k2
Prkag2
Ripk1
Rps6ka3
Scyl2
Sik1
Sik2
Sik3
Smg1
Trpm7

Supplementary Figures Legends:

Supplementary Fig. S1. Schematic workflow of SILAC phosphoproteomic analysis. Three replicates of samples were analyzed, including one label-swap measurement. For detailed description, see Supplementary methods.

Supplementary Fig. S2. Significantly regulated phosphorylation sites and phosphorylation pattern of phosphosites. (A) Phosphosites with localization probability >0.75 divided by amino acid (%). (B) Volcano plots showing reproducibility of phosphosite changes. Log2 values of normalized SILAC ratios are on x-axis, -log10 of p-values obtained from one-sample Student t-test (against 0) are on y-axis. Red points correspond to the phosphosites, which were significantly regulated (GMRT test, see Supplementary Methods). Only phosphosites, which were quantified in all three replicates of the respective experimental condition (time point and toxin-/toxoid-treatment type, Figure S1) were considered for plots. (C) Numbers of significantly regulated phosphorylation sites for each condition.

Supplementary Fig. S3. Scatter plots of all phosphosites identified in all three replicates for wild-type CyaA and CyaA-AC$^-$ toxoid-treated cells. (A) Significantly regulated phosphosites (red) found for cells exposed for 10 minutes to 100 ng/ml of the CyaA-AC$^-$ toxoid. (B) Significantly regulated phosphosites (red) found for cells treated for 10 minutes with 100 ng/ml of CyaA. (C) Significantly regulated phosphosites (red) found for cells treated for 30 minutes with 100 ng/ml of CyaA-AC-toxoid. (D) Significantly regulated phosphosites (red) found for cells for exposed for 30 minutes to 100 ng/ml of CyaA.

Supplementary Fig. S4. Dynamics of Erk1 phosphorylation status (T203/Y205) changes in BMDCs treated with wild-type CyaA or the CyaA-AC$^-$ toxoid. (A) Representative Western blot showing changes in phosphorylation status of Thr203/Tyr205 residues in the activation loop of p44/p42 Erk1/2 kinases of three independent biological replicates. (B) Relative quantification of changes in phosphorylation status of Thr203/Tyr205 in p44/Erk1 compared to negative control (buffer). (C-D) Full-length immunoblots. TUC stands for TUC buffer (50 mM Tris-HCl, 8 M urea, 2 mM $CaCl_2$, pH 8), which was used for toxin/toxoid dilution.

Supplementary Fig. S5. Best-scoring MS/MS spectrum of phosphorylated peptide RPpSLGAVER from CyaA processed and annotated by MaxQuant. Phosphopeptide carrying serine 393 of CyaA and phosphorylated by host cell was detectable in BMDC lysates digests following the first 10 min of treatment by both CyaA and CyaA-AC^{-}.

Supplementary Fig. S6: Relative quantification of phosphorylation. Results for probing with (A) anti-p-PRAS40 (Thr247), (B) anti-p-mTOR (Ser2448) and (C) anti-p-4E-BP1 (Ser64) antibodies. The values were are the mean of three biological replicates relative to the negative control – TUC-treated cells. TUC stands for TUC buffer (50 mM Tris-HCl, 8 M urea, 2 mM $CaCl_2$, pH 8), which was used for toxin/toxoid dilution.

Supplementary Fig. S7. Full-length blots for Figure 3 showing changes in phosphorylation status of members of mTOR signaling pathway and of cytoskeletal regulator VASP. Immunoblots for analysis of phosphorylation status of (A-C) threonine 247 residue of PRAS40, (D-E) serine 2448 residue of mTOR, (F-H) serine 64 residue of 4E-BP1 and (I-J) serine 235 residue of VASP (residues are numbered according to mouse gene sequences). TUC stands for TUC buffer (50 mM Tris-HCl, 8 M urea, 2 mM $CaCl_2$, pH 8), which was used for toxin/toxoid dilution.

Supplementary Fig. S8. Phosphorylation status Akt kinase after treatment with wild-type CyaA or the CyaA-AC^{-} toxoid. (A) Phosphorylation status of Thr308 and Ser473 residues of Akt. (B) and (C) Full-length blots for immunostainings with α-p-Akt Thr308 and α-pan Akt antibodies, respectively. (D) and (E) Full-length blots for immunostainings with α-p-Akt Ser473 and α-pan Akt antibodies, respectively (residues number according to mouse gene sequences). TUC stands for TUC buffer (50 mM Tris-HCl, 8 M urea, 2 mM $CaCl_2$, pH 8), which was used for toxin/toxoid dilution.

Supplementary figures:

Supplementary Figure S1

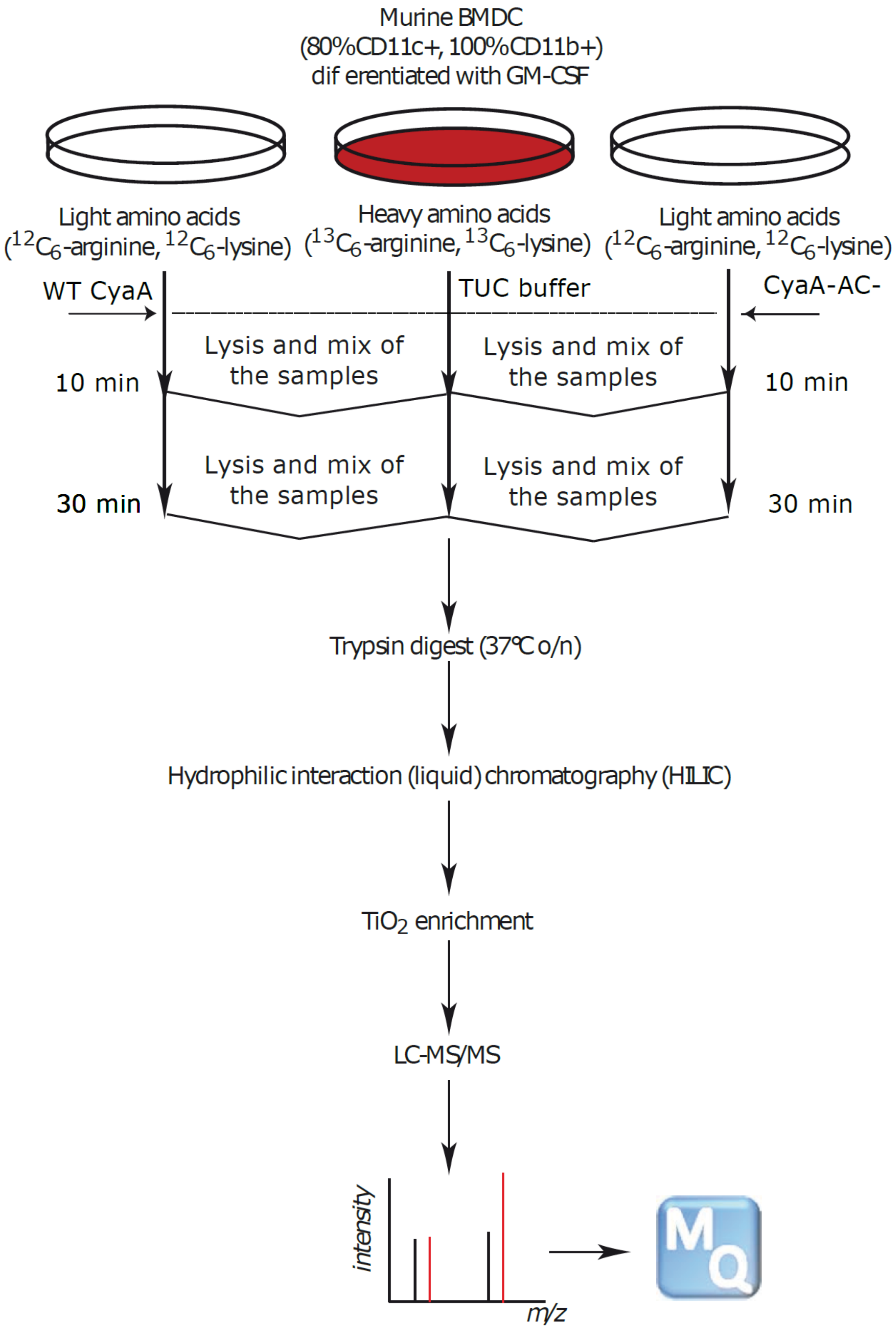

Supplementary Figure S2A

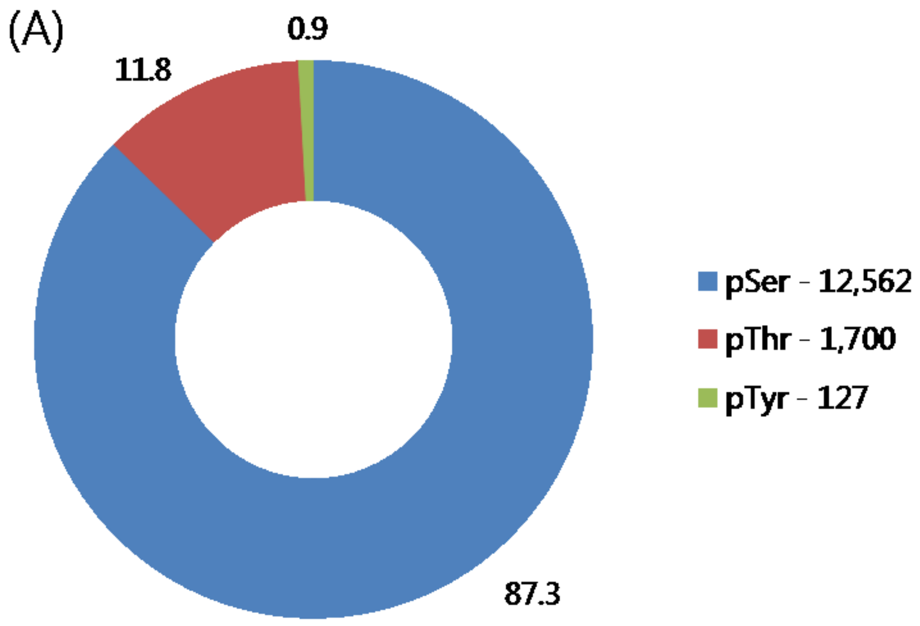

Supplementary Figure S2B

(B)

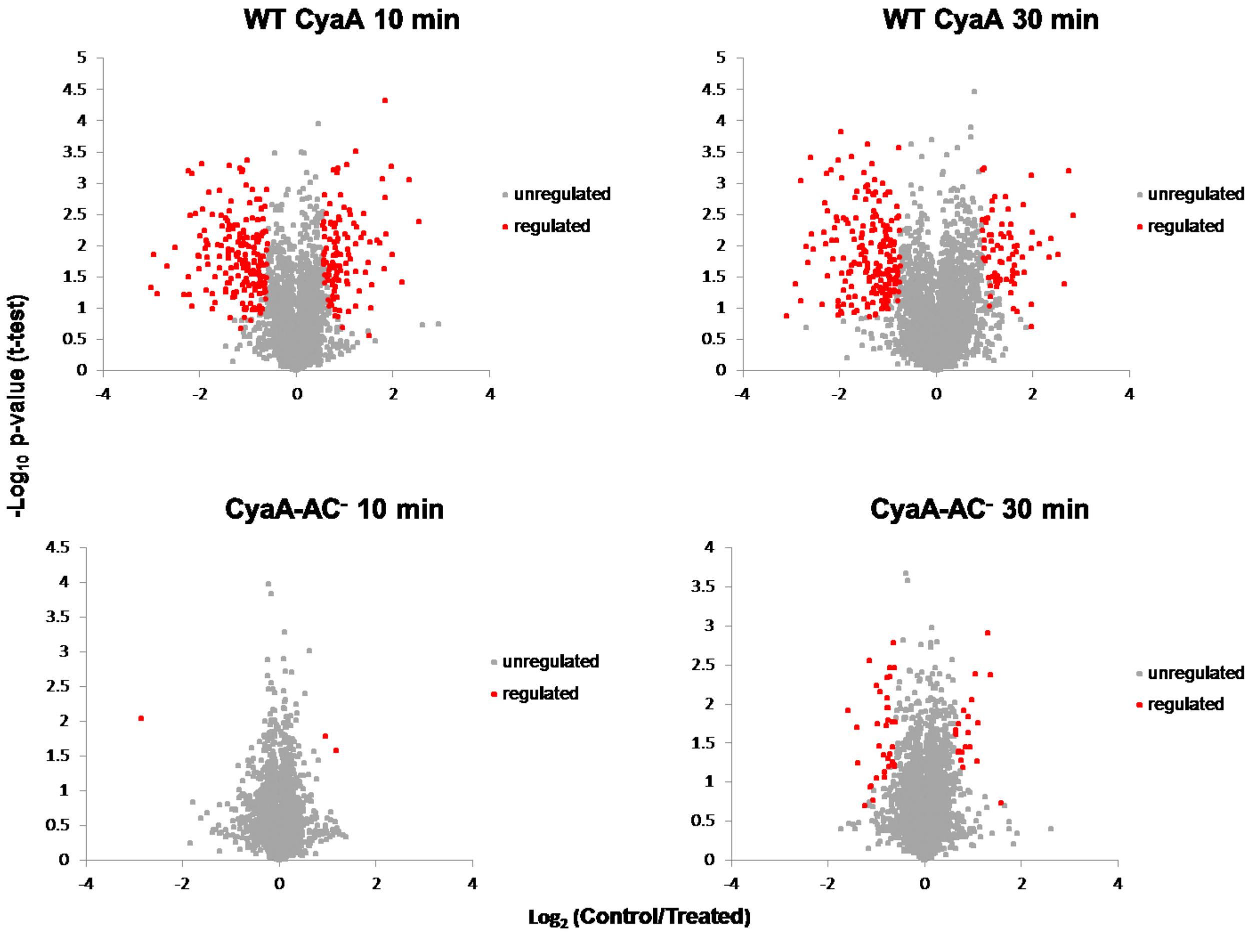

Supplementary Figure S2C

(C)

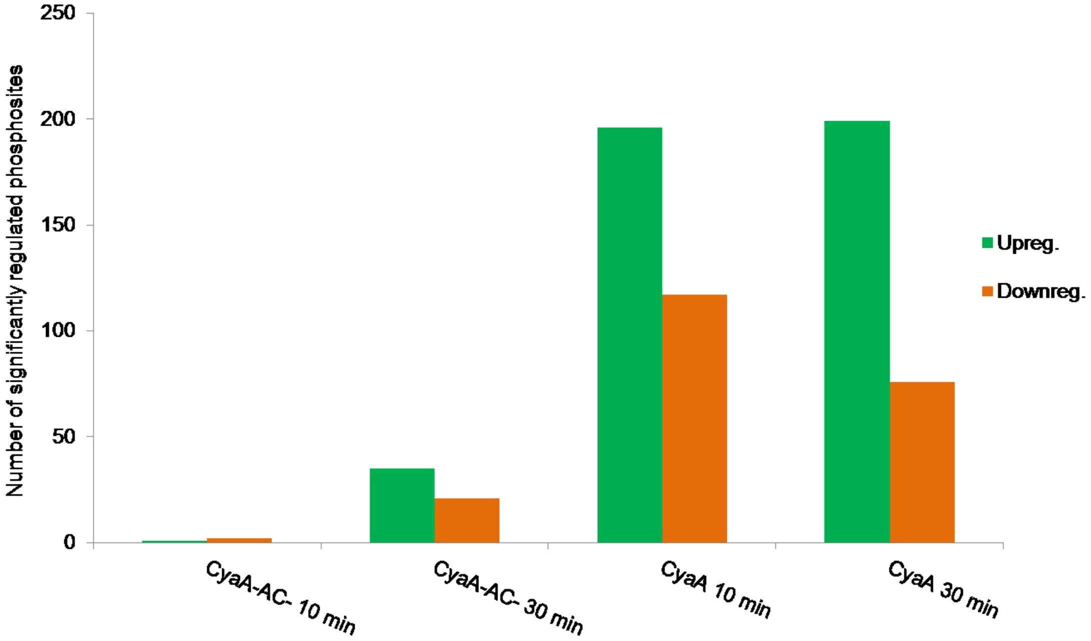

Supplementary Figure S3A

(A)

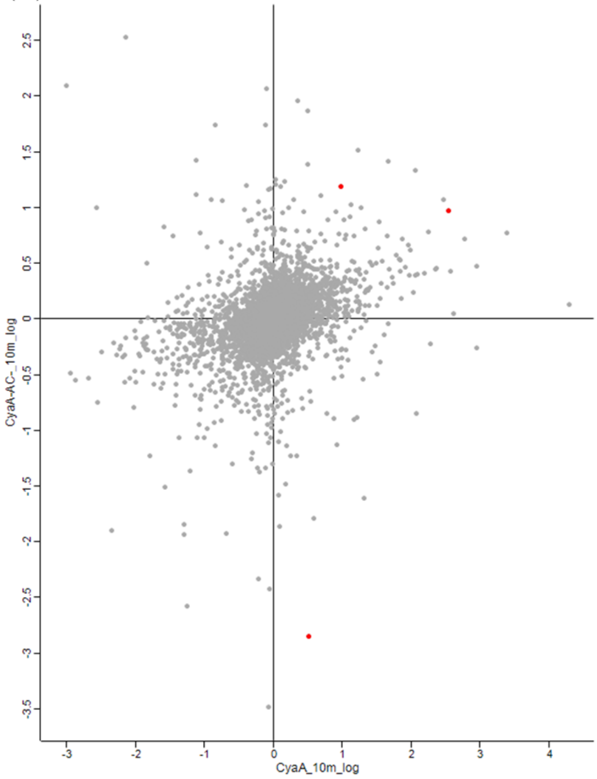

Supplementary Figure S3B

(B)

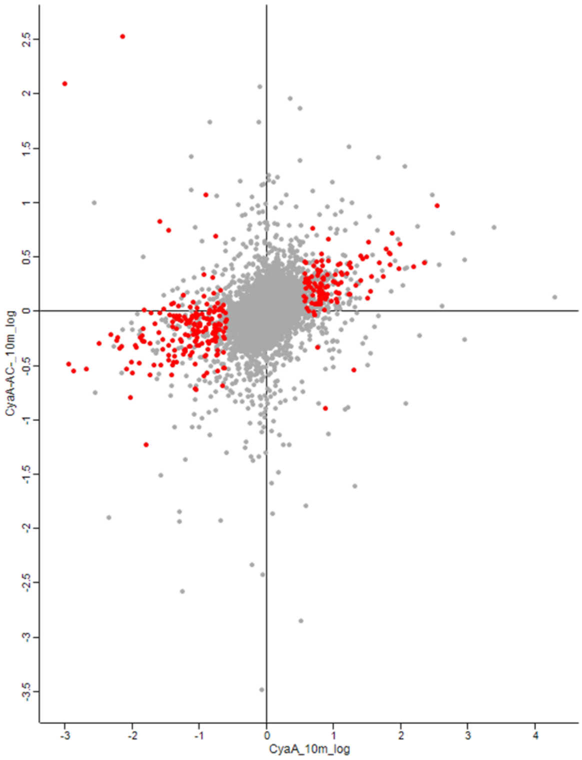

Supplementary Figure S3C

(C)

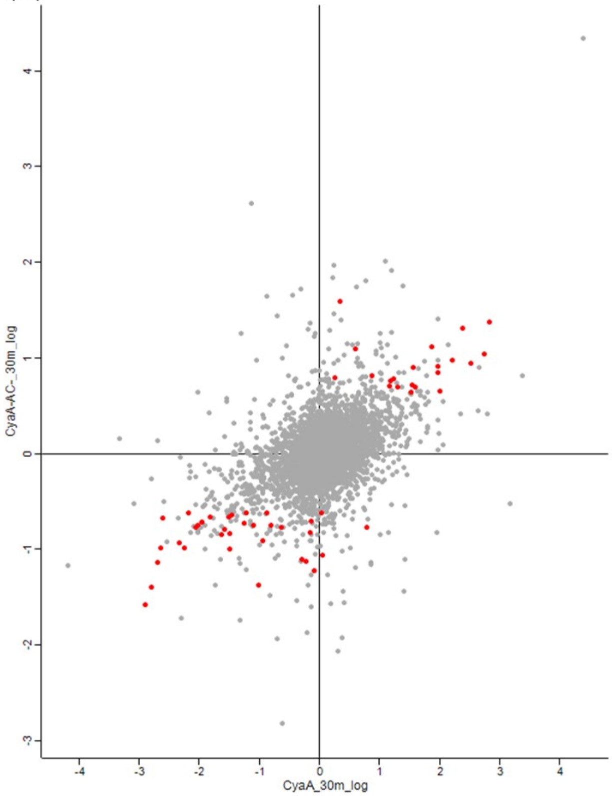

Supplementary Figure S3D

(D)

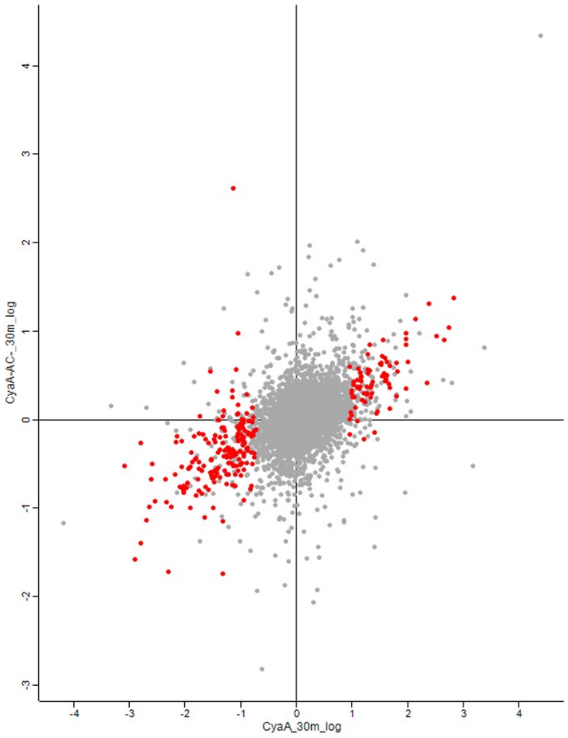

Supplementary Figure S4A

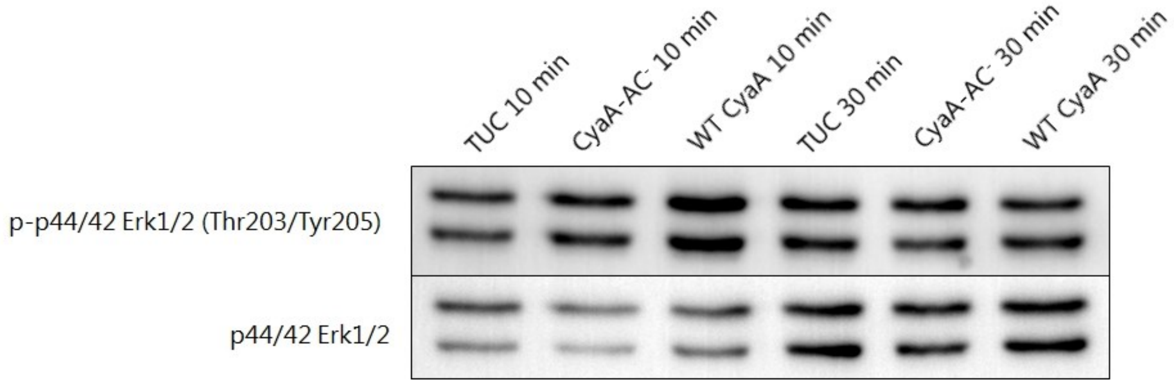

Supplementary Figure S4B

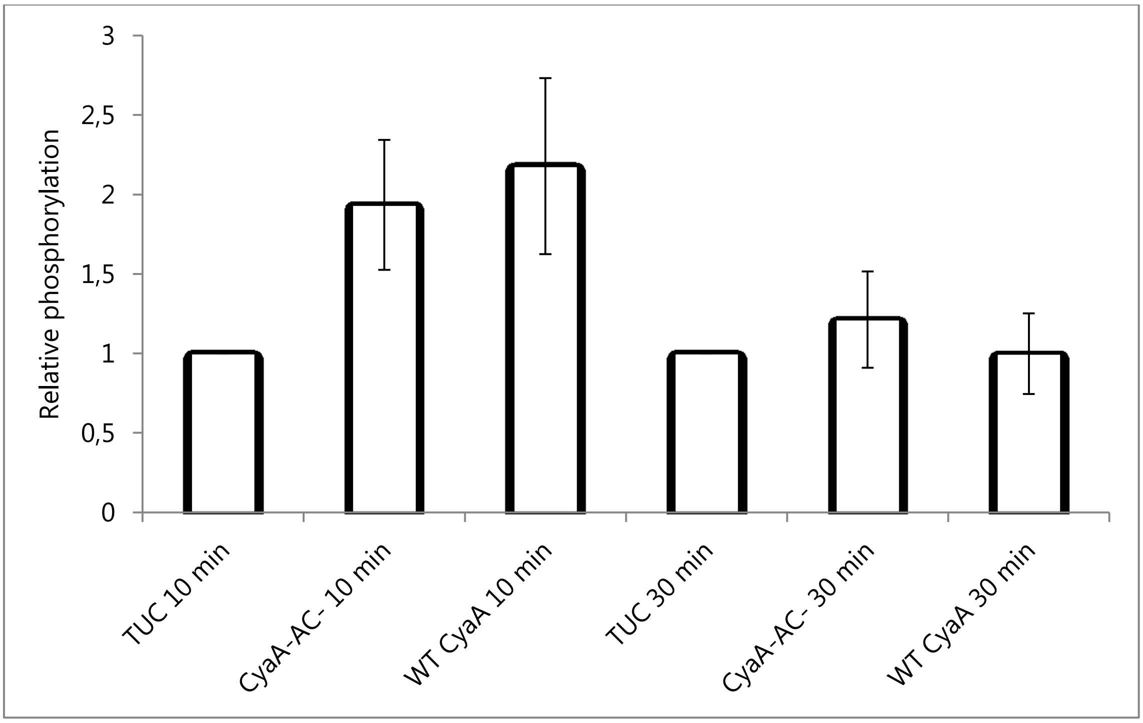

Supplementary Figure S4C

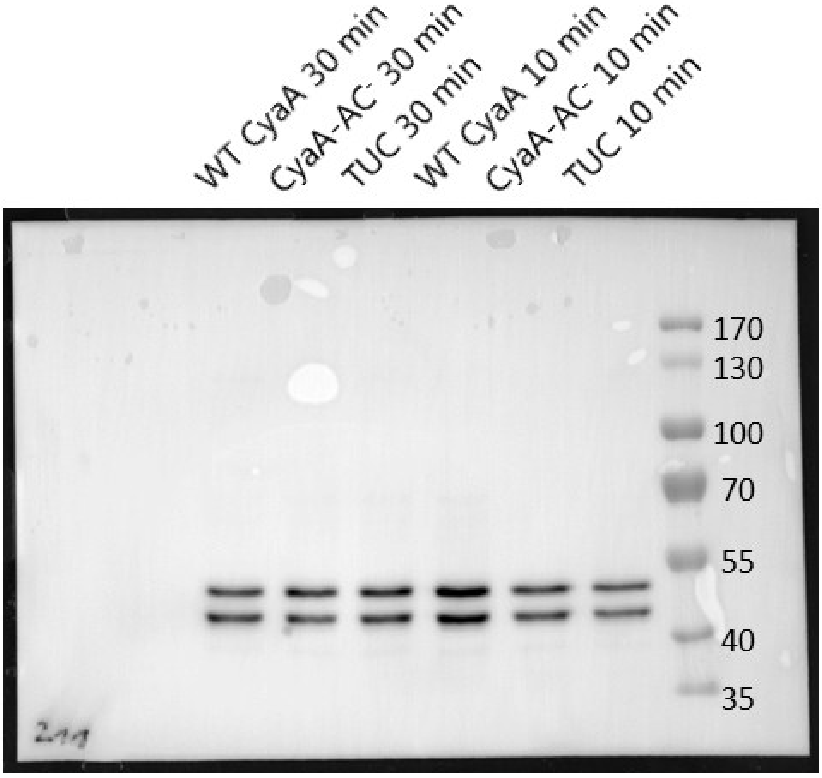

p-p44/42 Erk1/2 (Thr203/Tyr205) 7.5% SDS-PAGE

Supplementary Figure S4D

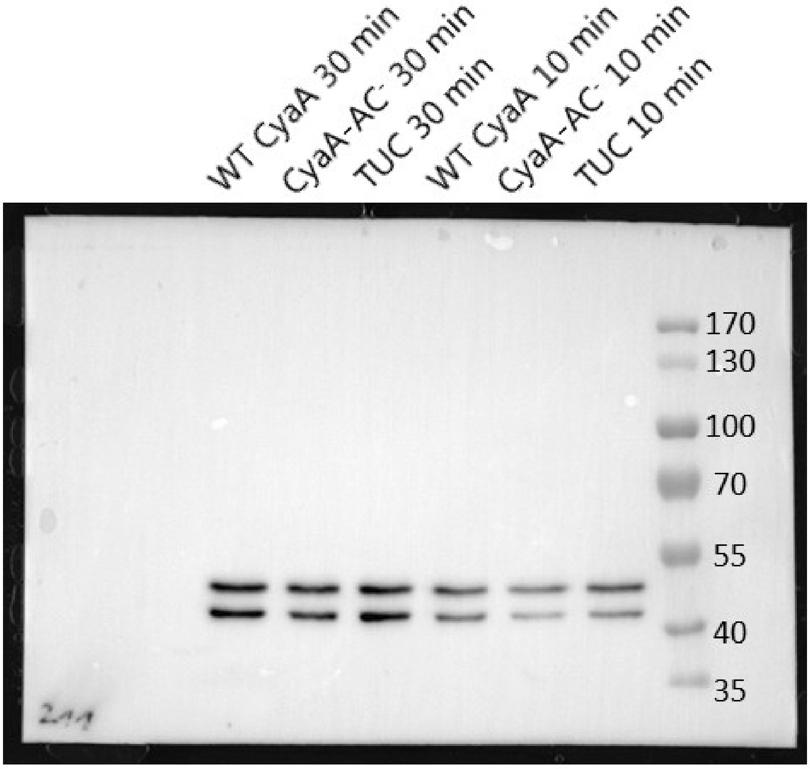

p44/42 Erk1/2 7.5% SDS-PAGE

Supplementary Figure S5

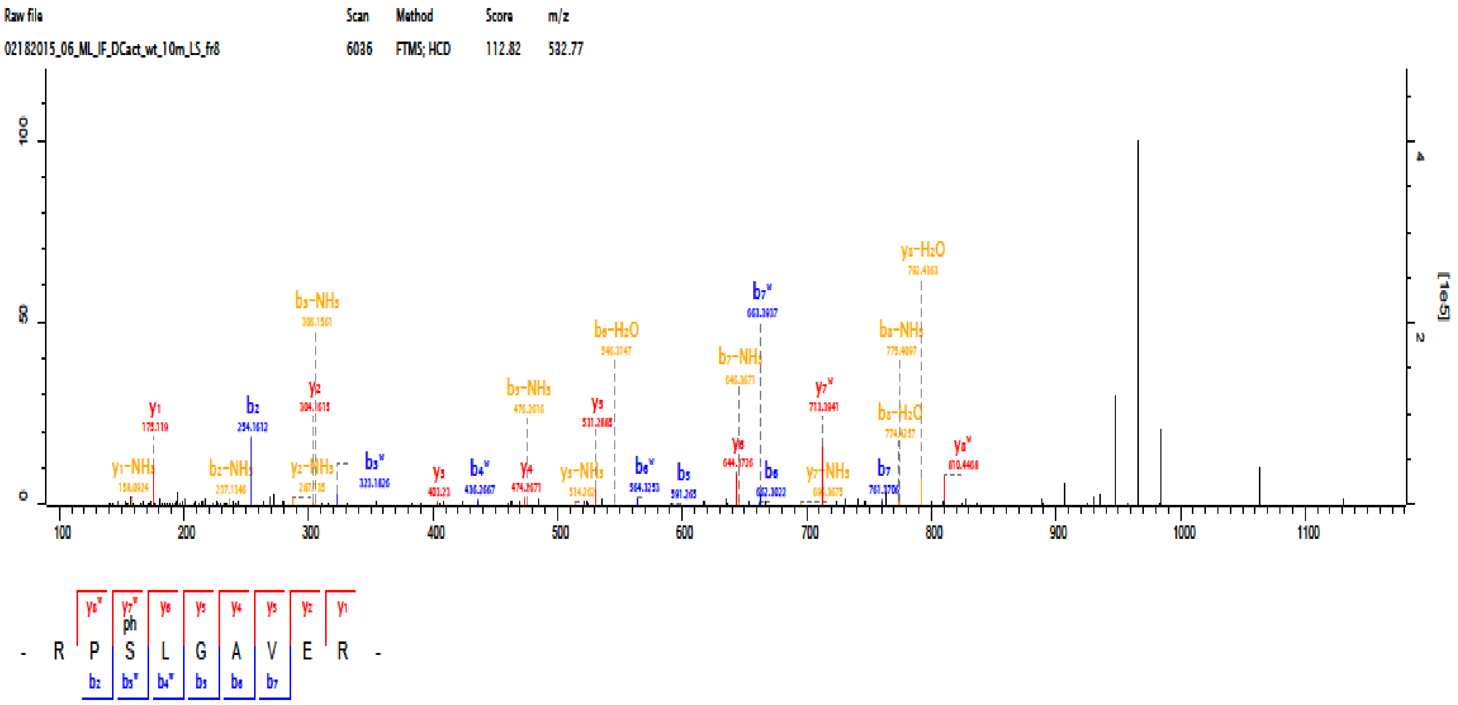

Supplementary Figure S6A

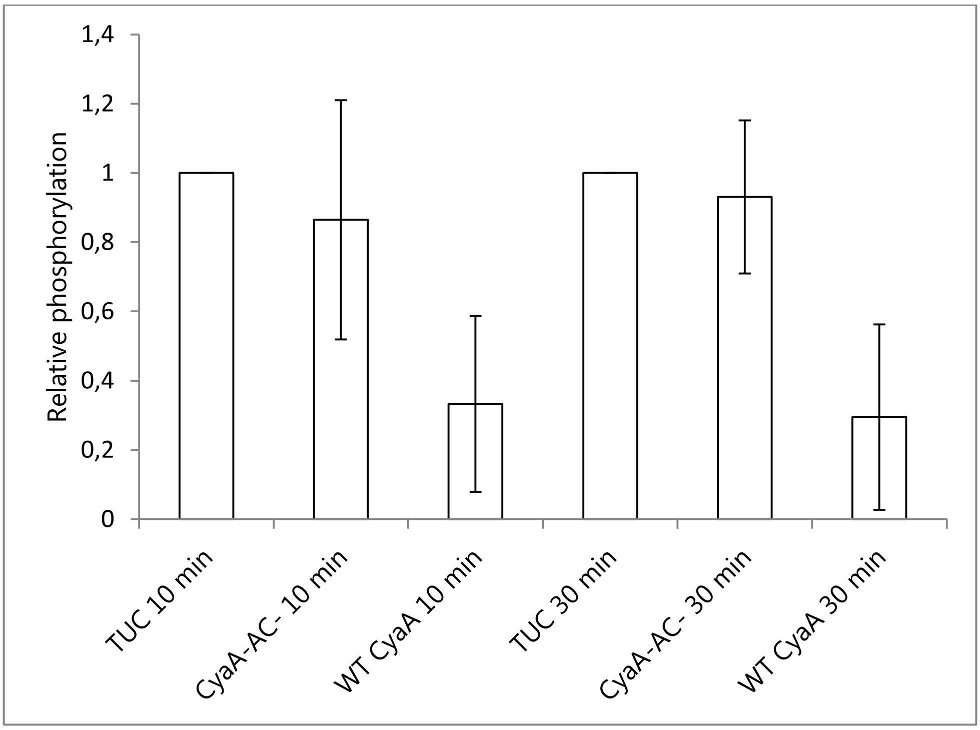

Supplementary Figure S6B

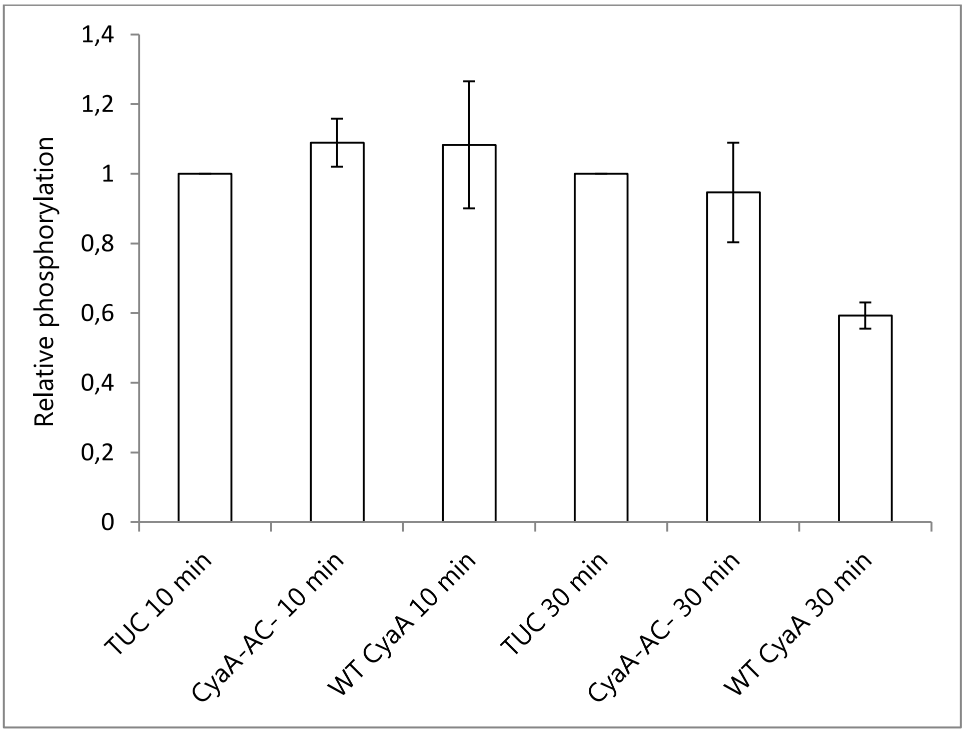

Supplementary Figure S6C

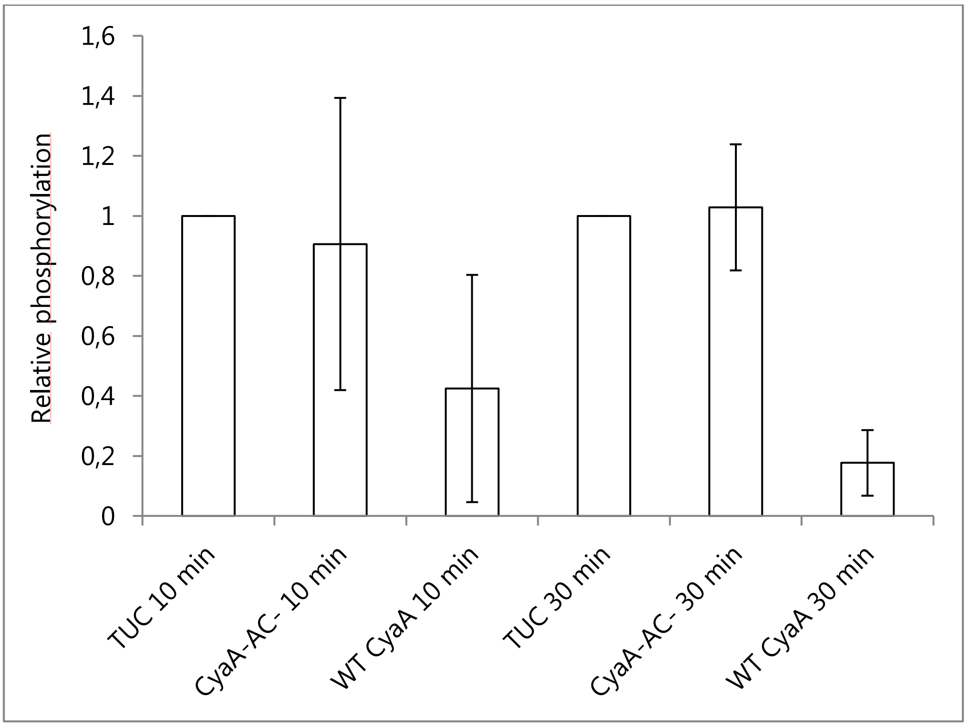

Supplementary Figure S7A

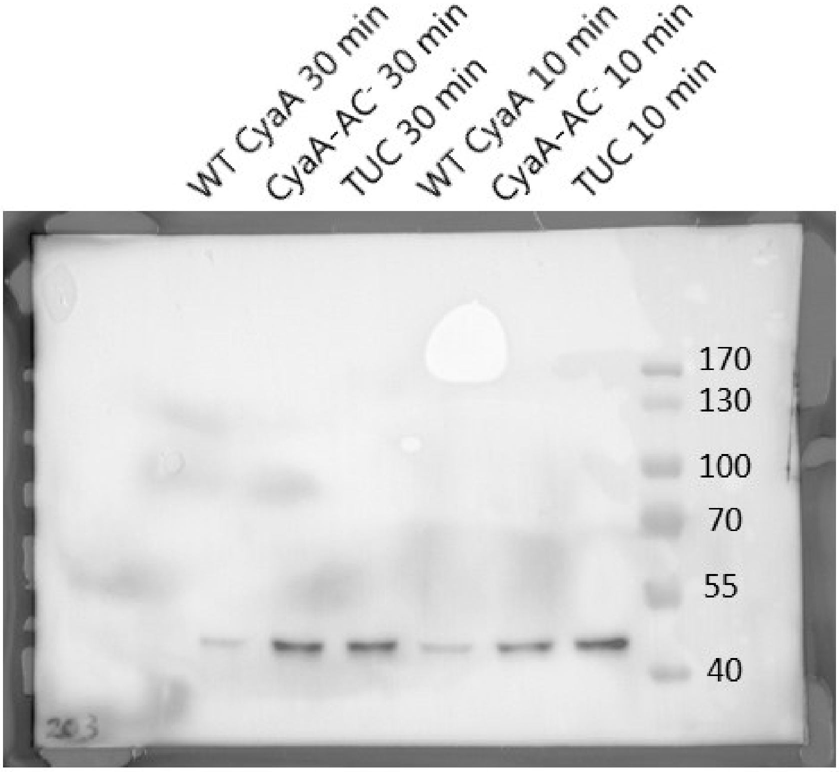

p-PRAS40 (Thr247) 7.5% SDS-PAGE

Supplementary Figure S7B

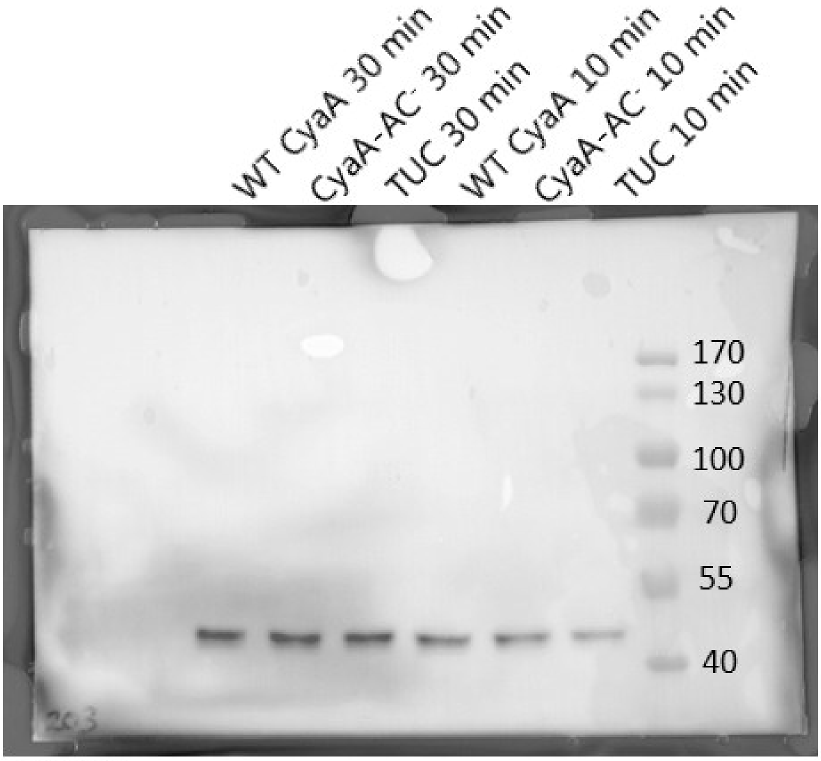

PRAS40 7.5% SDS-PAGE

Supplementary Figure S7C

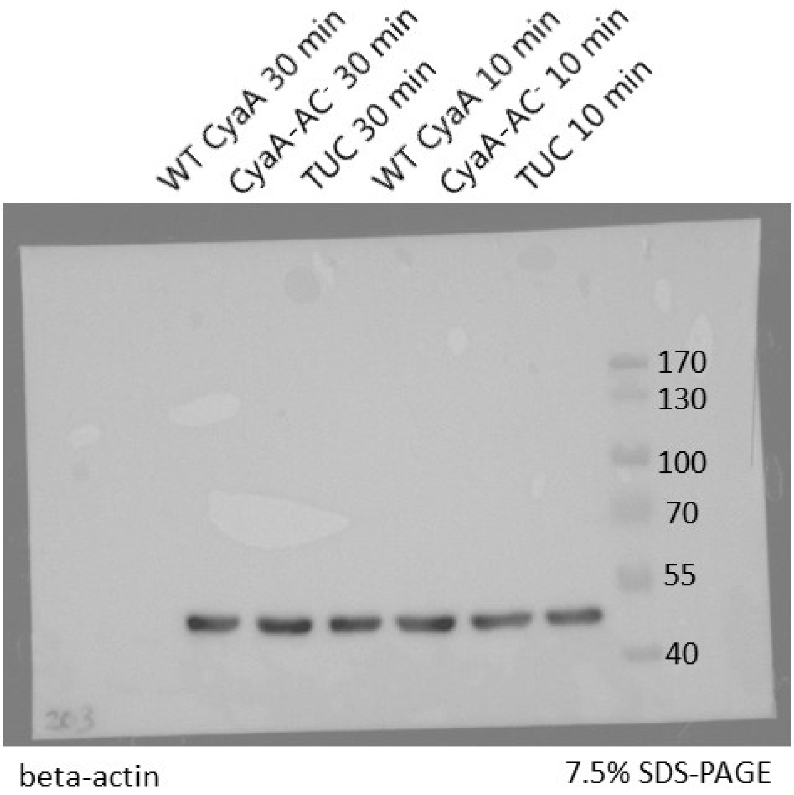

Supplementary Figure S7D

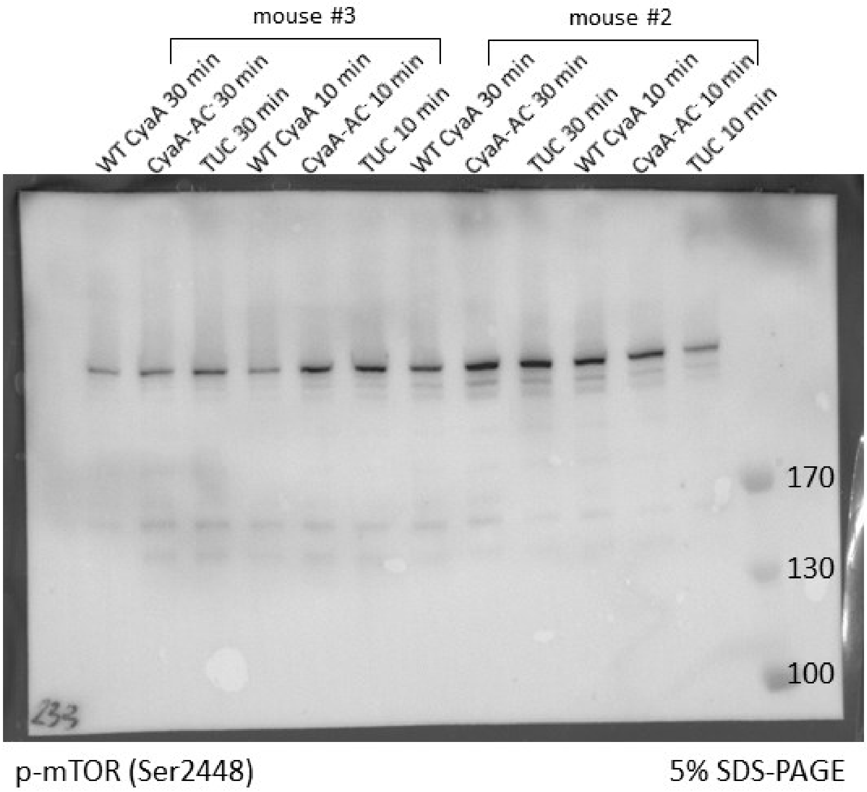

Supplementary Figure S7E

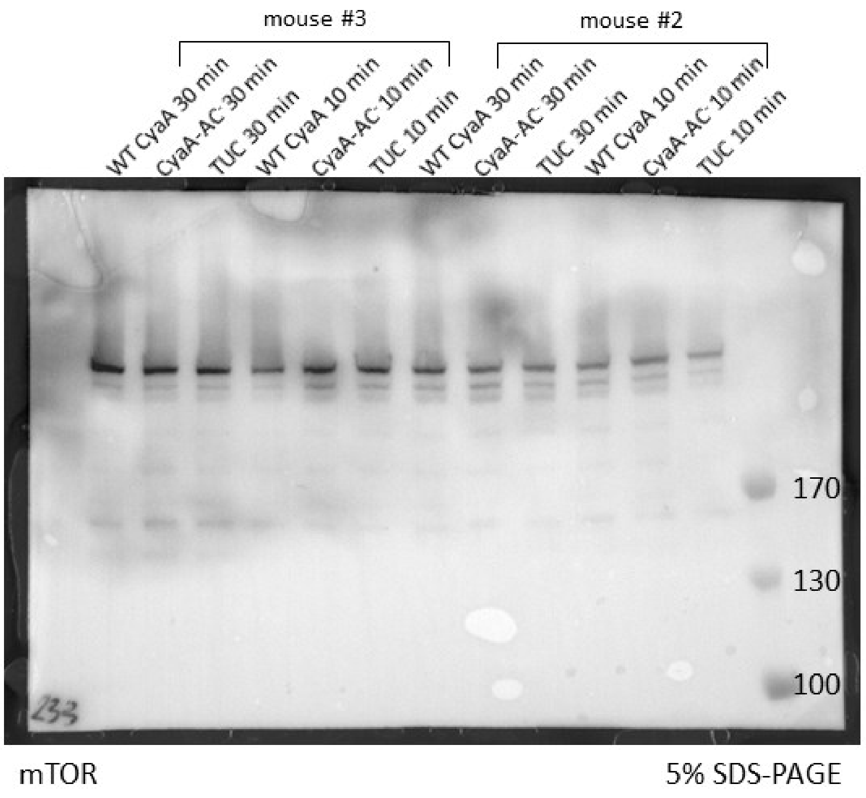

Supplementary Figure S7F

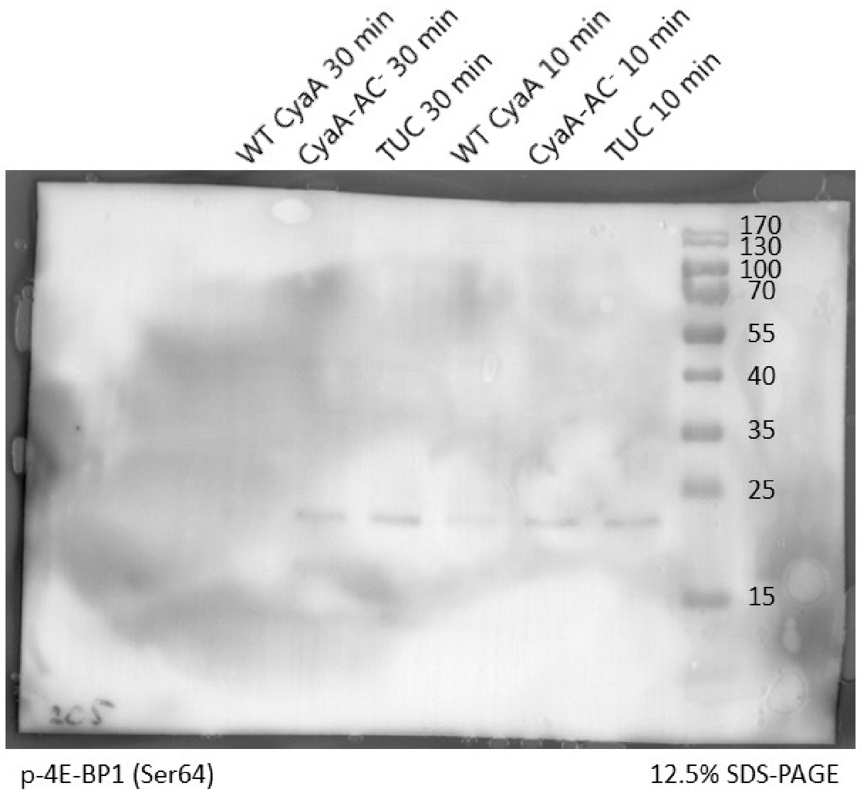

Supplementary Figure S7G

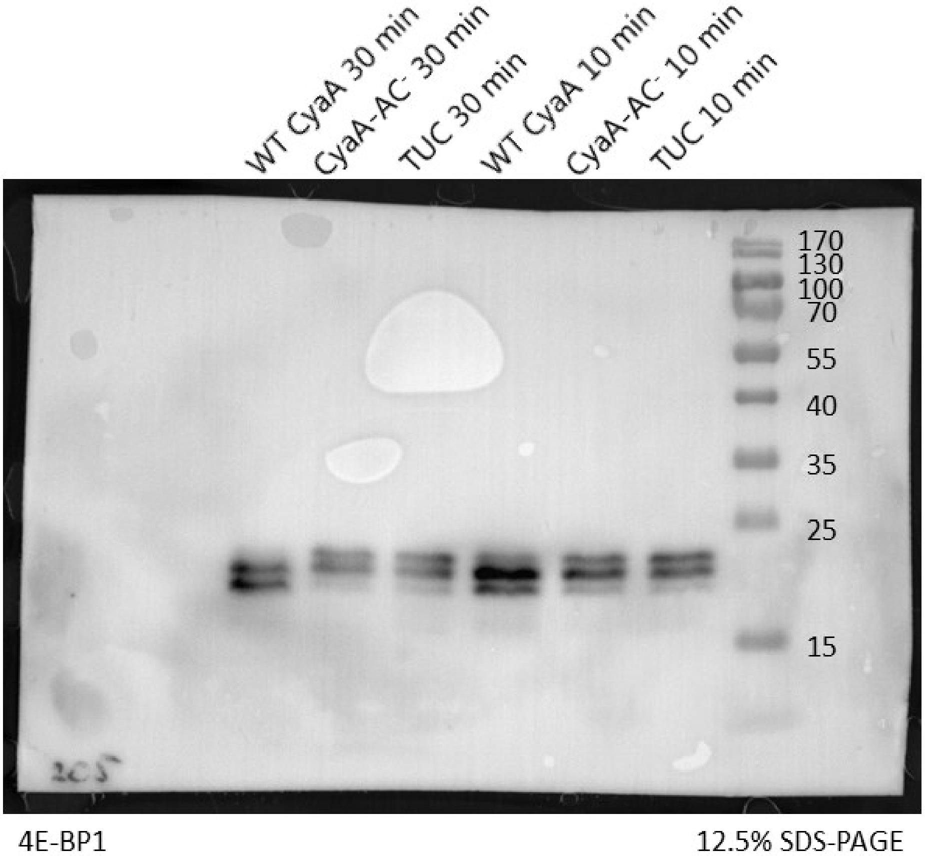

Supplementary Figure S7H

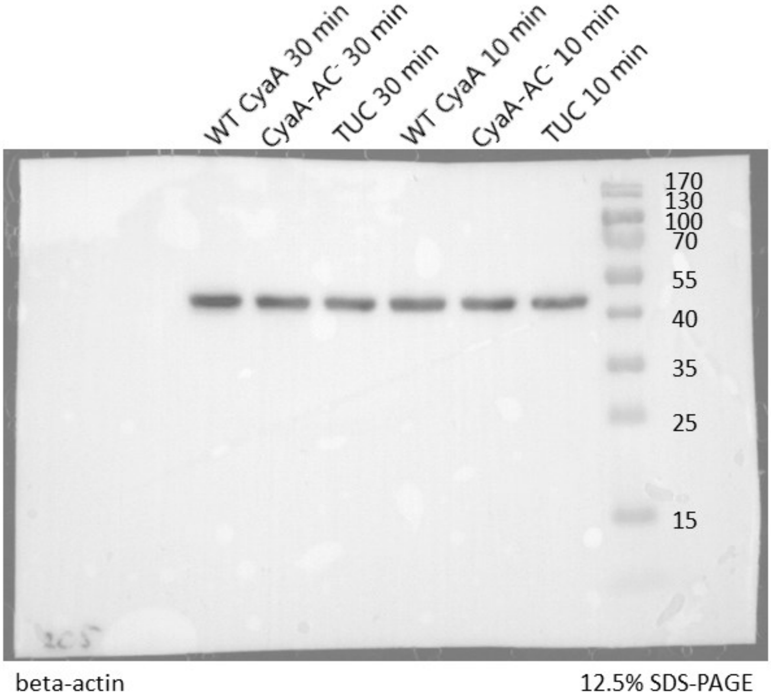

Supplementary Figure S7I

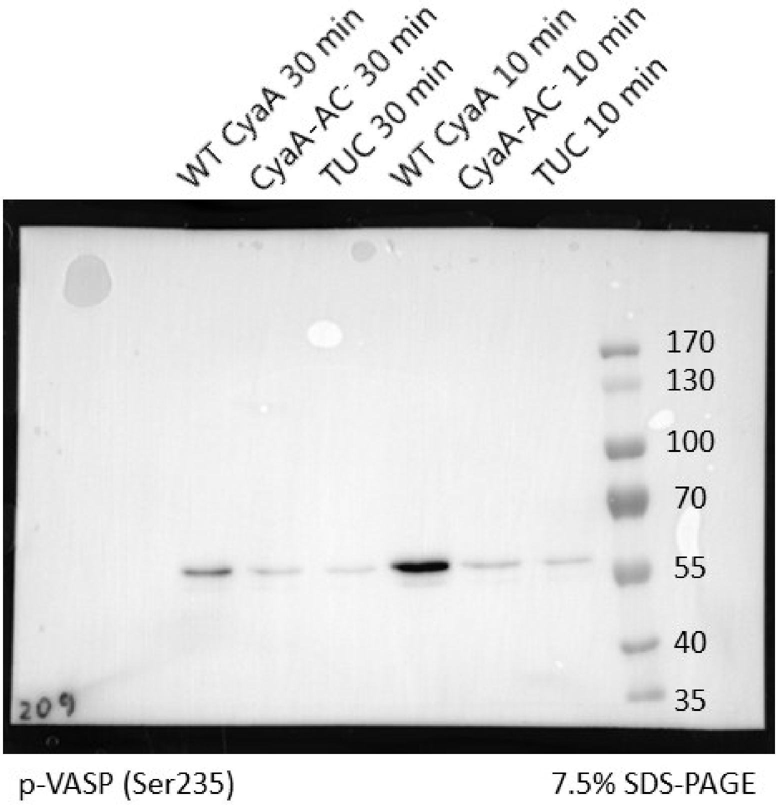

Supplementary Figure S7J

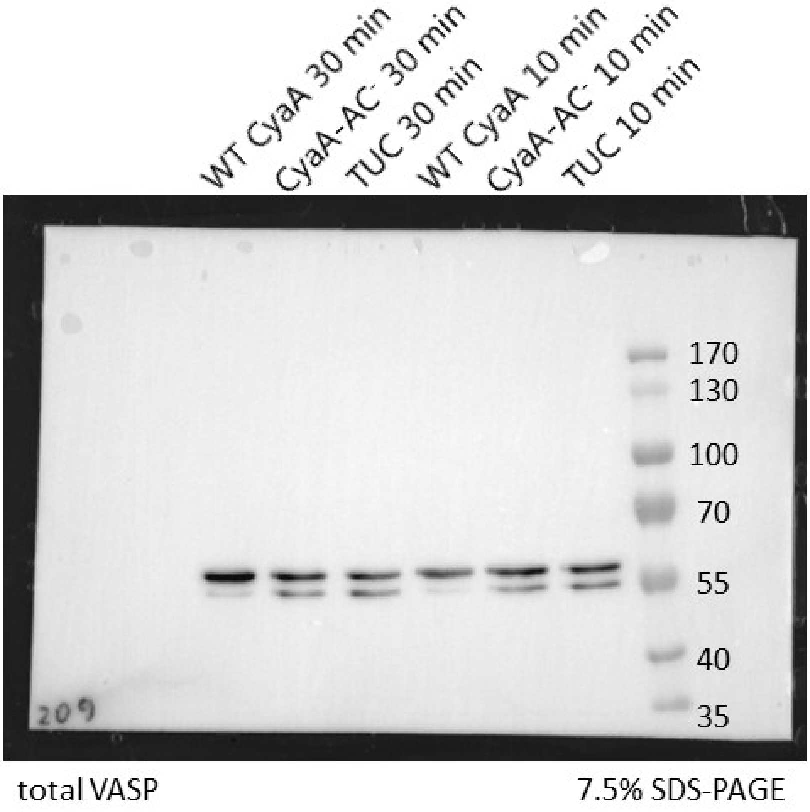

Supplementary Figure S8A

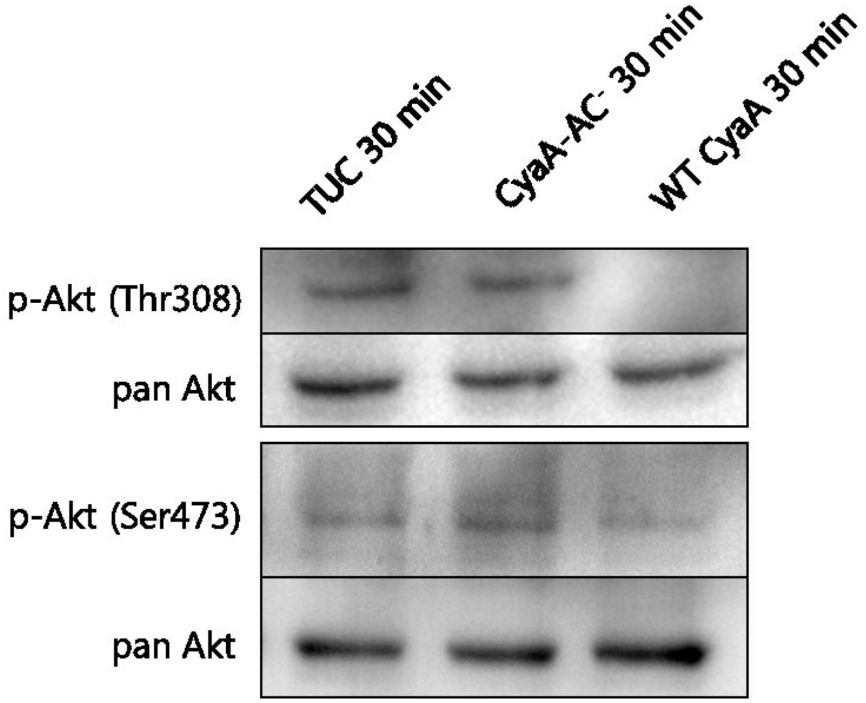

Supplementary Figure S8B

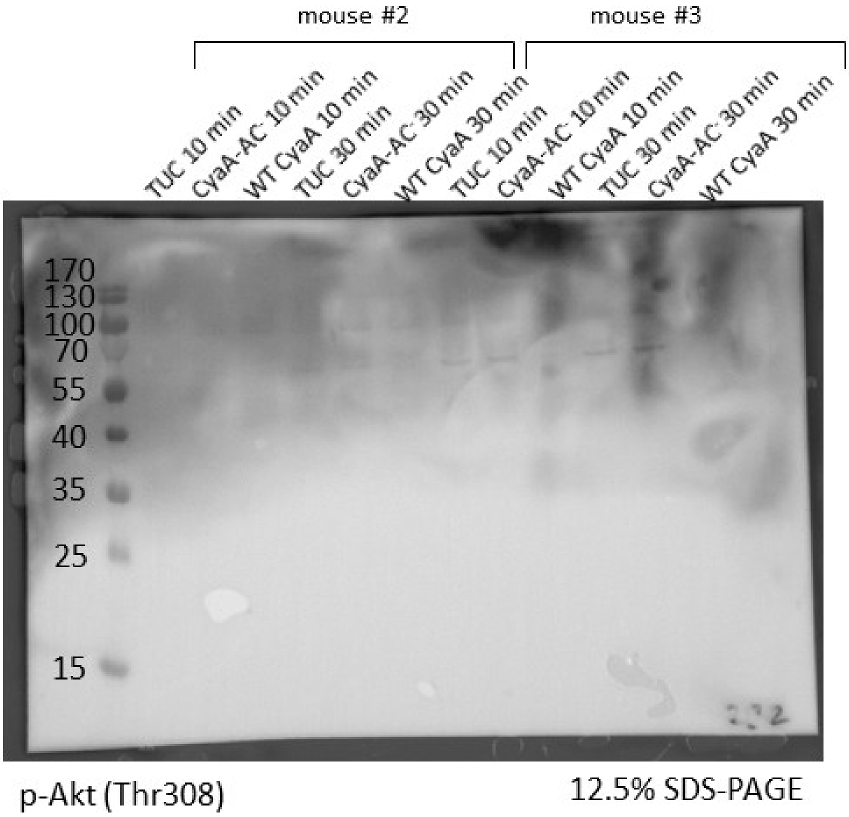

Supplementary Figure S8C

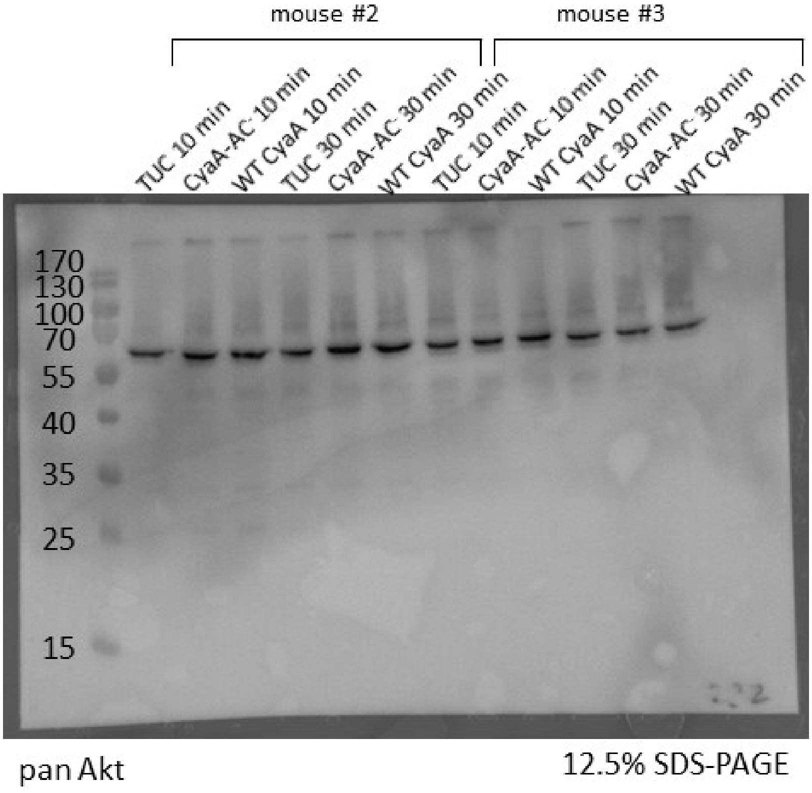

Supplementary Figure S8D

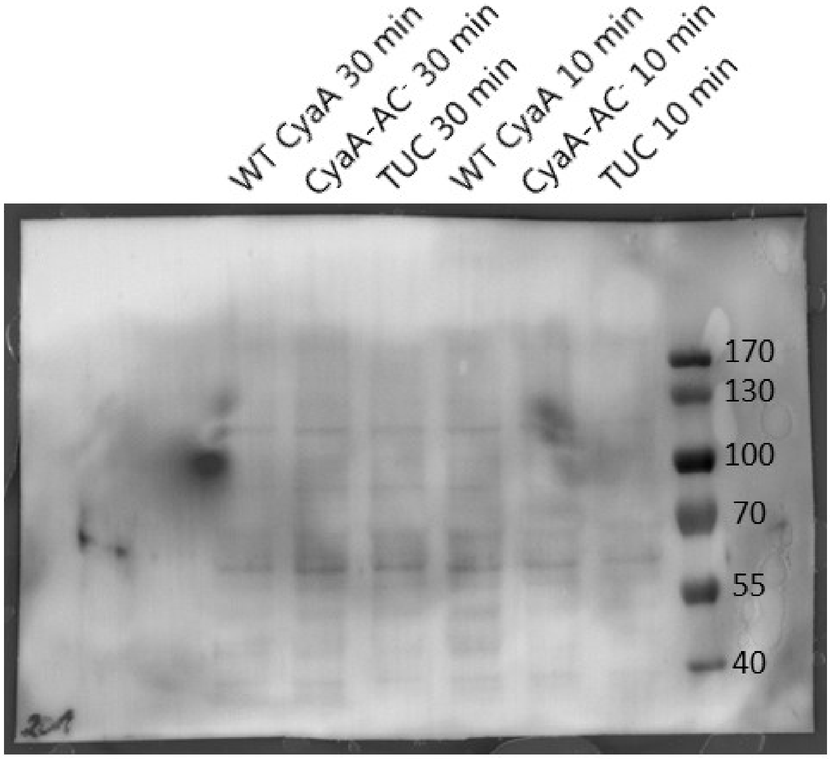

p-Akt (Ser473) 7.5% SDS-PAGE

Supplementary Figure S8E

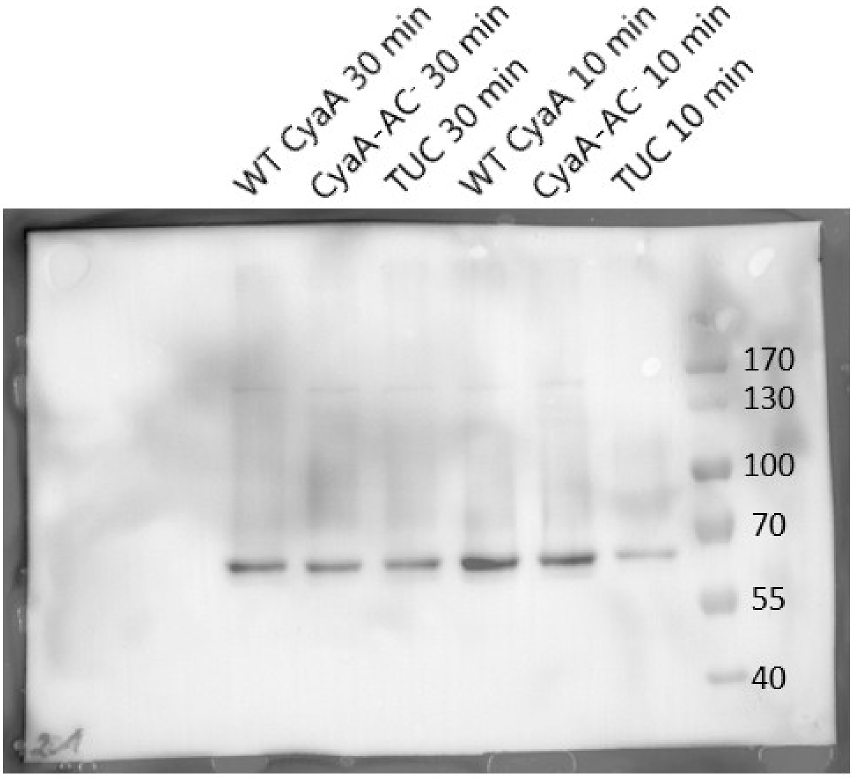

pan Akt 7.5% SDS-PAGE
